# Supplementary material for: Programmable mammalian translational modulators by CRISPR-associated proteins
Source: Nat Commun. 2023 Apr 19;14:2243. doi: 10.1038/s41467-023-37540-7 (PMC10115826; doi:10.1038/s41467-023-37540-7)
Supplement: Supplementary file 1 — Supplementary Information [file 41467_2023_37540_MOESM1_ESM.pdf]

Supplementary Information

Programmable mammalian translational modulators by CRISPR-associated proteins

Shunsuke Kawasaki[1,4], Hiroki Ono[1,2,4], Moe Hirosawa[1,4], Takeru Kuwabara[3], Shunsuke Sumi[1,2], Suji Lee[1,2], Knut Woltjen[1] & Hirohide Saito[1,2]

1 Department of Life Science Frontiers, Center for iPS Cell Research and Application, Kyoto University, 53 Kawahara-cho, Shogoin, Sakyo-ku, Kyoto 606-8507, Japan.
2 Graduate School of Medicine, Kyoto University, Yoshida-Konoe-cho, Sakyo-ku, Kyoto 606-8501, Japan.
3 Faculty of Medicine, Kyoto University, Yoshida-Konoe-cho, Sakyo-ku, Kyoto 606-8501, Japan.
4 These authors contributed equally: Shunsuke Kawasaki, Hiroki Ono, Moe Hirosawa.

*Corresponding authors: Shunsuke Kawasaki, Hirohide Saito.
**Lead contact: Hirohide Saito (saitou.hirohide.8a@kyoto-u.ac.jp).

Contents

Supplementary Figure 1| Schematic diagrams of experimental procedures in plasmid transfection.
Supplementary Figure 2| Original validation data of each Cas-responsive OFF switch related to Figure 1E.
Supplementary Figure 3| SpCas9-responsive reporter repression is independent of transcriptional regulations.
Supplementary Figure 4| The performance of SpCas9-responsive switch in RNA transfection.
Supplementary Figure 5| The performance of SpCas9-responsive switch for the iPSC genome integrated Cas9.
Supplementary Figure 6| The effects of tandem insertion of sgRNAs.
Supplementary Figure 7| The validation of the position of sgRNA insertion.
Supplementary Figure 8| Original validation data of each Cas-responsive ON switch related to Figure 2B.
Supplementary Figure 9| Correlation of the performance in Cas-responsive OFF switches and ON switches.
Supplementary Figure 10| Effects of RNase activity on the AsCas12a-responsive switch.
Supplementary Figure 11| Relationships in verified parameters.
Supplementary Figure 12| The translational repression with auto-assemble split-Cas9.
Supplementary Figure 13| Orthogonality heat-map among representative Cas-responsive OFF switches related to Figure 3A.
Supplementary Figure 14| Fluorescence cell images and heat-map of the 25 × 25 orthogonality matrix of representative Cas proteins and Cas-responsive ON switches.
Supplementary Figure 15| Extraction of orthogonal pairs in Cas proteins and Cas-responsive OFF and ON switches related to Figure 4.
Supplementary Figure 16| Phylogenetic tree of Cas9 sgRNA used in OFF switch orthogonality.
Supplementary Figure 17| Simultaneous regulation of translational activation and repression with SaCas9.
Supplementary Figure 18| Cell phenotype control with AND gate circuits related to Figure 6E, F.

Supplementary Text 1| Investigating the strategies for improving Cas-responsive switches.
Supplementary Table 1| Information of Cas proteins used in this study.
Supplementary Table 2| Information about the inserted RNA sequences and their translational repression efficiencies.
Supplementary Table 3| Components of each 60 AND gate tested in Figure 6.
Supplementary Table 4| Statistical analysis related to Figure 6F.
Supplementary Table 5| Key transfected plasmids used in this study.
Supplementary Table 6| Primers and template oligo DNA used for generating synthetic mRNAs.
Supplementary Table 7| Transfection tables of all experiments performed in this study.

Supplementary Sequences
Supplementary References

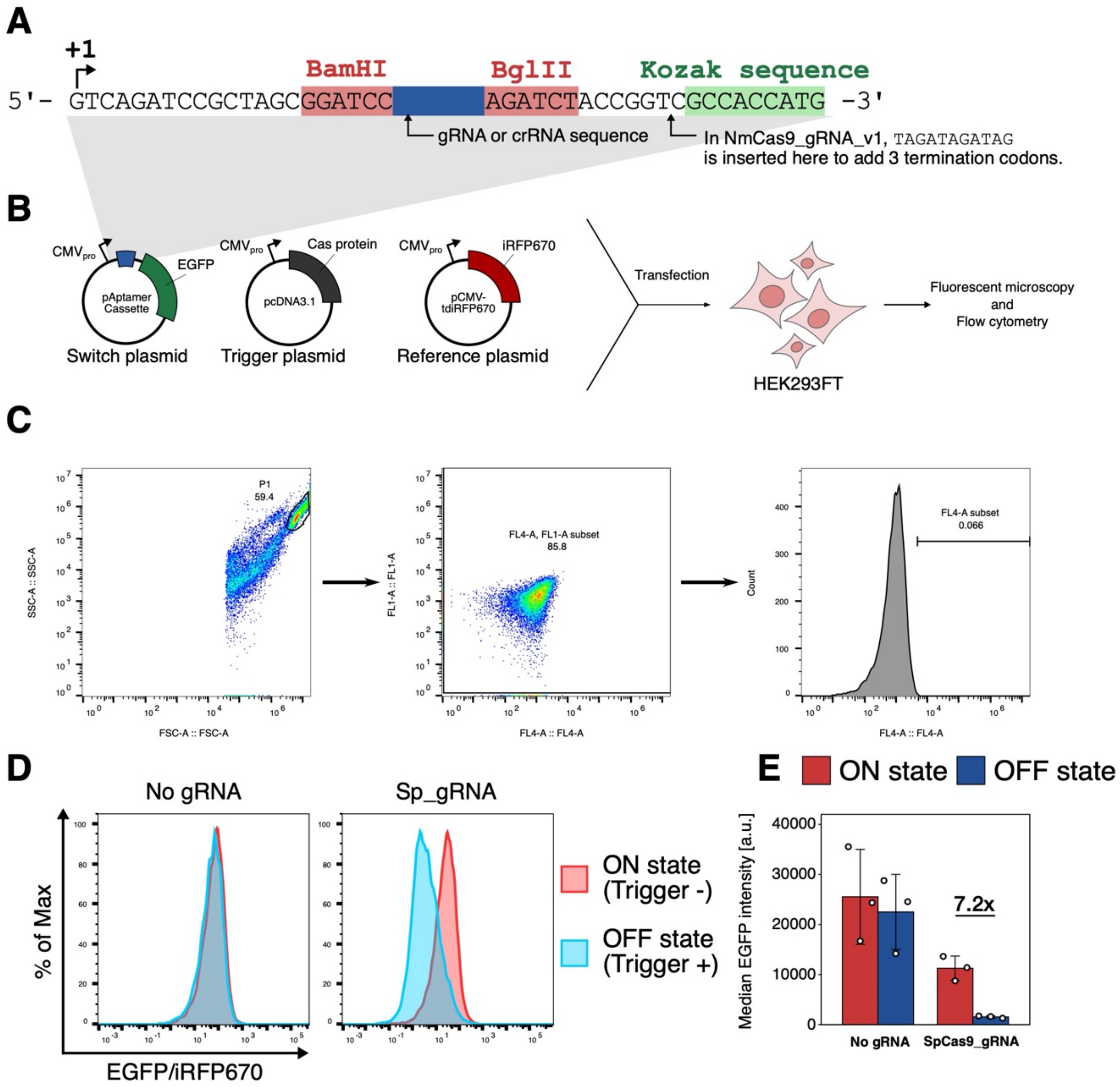

Supplementary Figure 1|

Schematic diagrams of experimental procedures in plasmid transfection.

(A) Illustration of the 5'-UTR regions in mRNA transcribed from switch plasmid. The flanking sequence of the inserted sgRNA is also shown. (B) Schematic diagram of our standard experimental workflow. Three plasmids (switch, trigger, and reference plasmid) were transfected into HEK293FT cells. Cells were analyzed by fluorescence microscopy and flow cytometer. (C) Gating procedures of flow cytometry. Flow cytometry datasets were analyzed using FlowJo version10.5.3 (see also Methods section). Live cells were gated in the forward scatter (FSC) versus side scatter (SSC) plot to eliminate debris. The remaining P1-positive events were plotted in the FL1-A (EGFP expression, Y-axis) versus FL4-A (iRFP670 expression, X-axis) and events on each axis were ruled out by the gate (FL4-A, FL1-A subset). The FL4-A subset gate, generated by using untransfected control samples, was used to define transfection-positive (FL4-positive) populations. (D) Representative histograms of expression level from the SpCas9-responsive EGFP-OFF switch with (blue) and without (red) the trigger SpCas9 plasmid. (E) Median EGFP expression levels in HEK293FT cells. Data are represented as the mean ± SD from three independent experiments. ON (trigger -) and OFF (trigger +) states are indicated in red and blue, respectively. We observed a similar tendency between the absolute EGFP expression levels and the Relative Intensities (See also Supplementary Figure 2). a.u.: arbitrary unit. Source data are provided as a Source Data file.

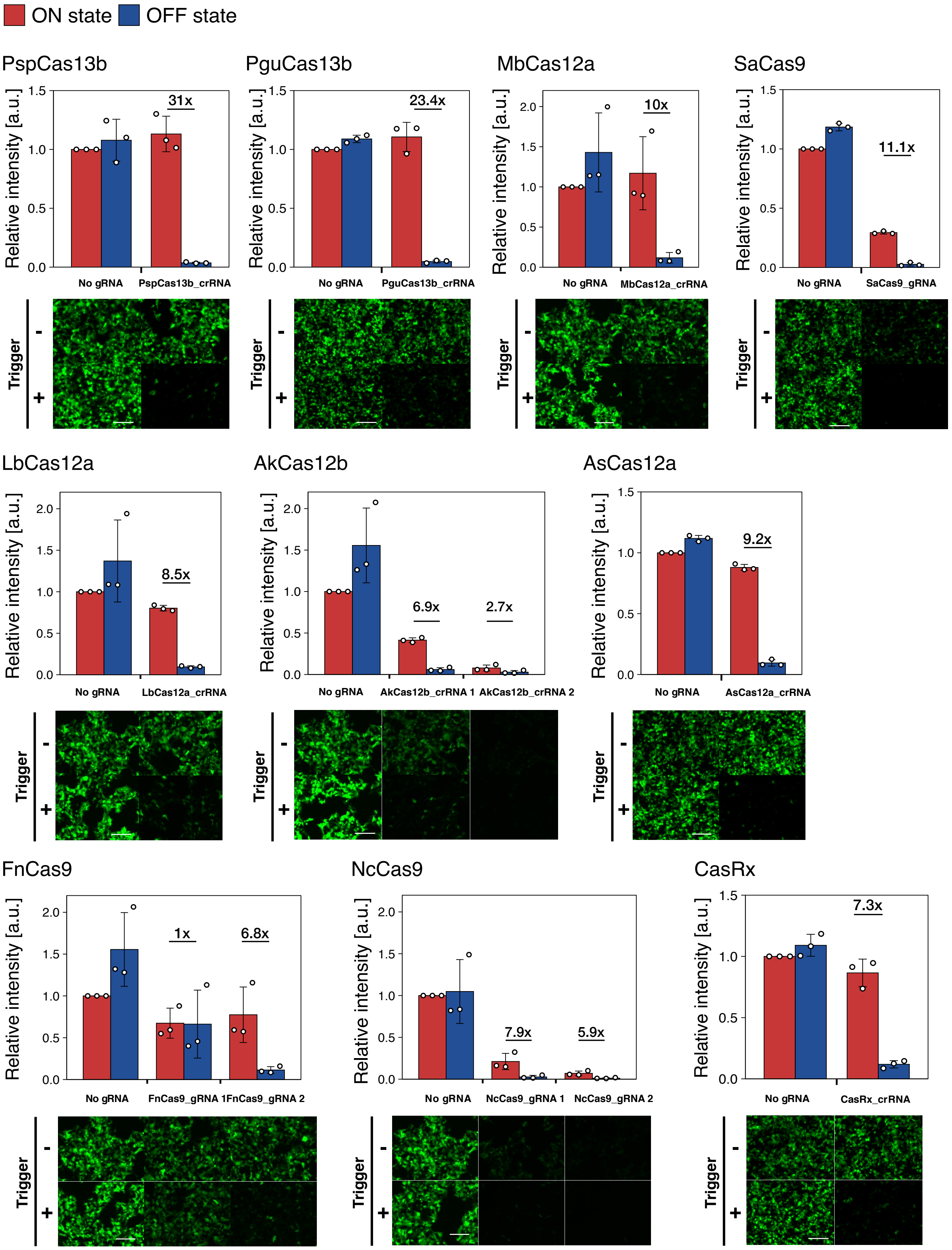
ON state
OFF state
PspCas13b
PguCas13b
MbCas12a
SaCas9
Relative intensity [a.u.]
31x
23.4x
10x
11.1x
No gRNA
PspCas13b_crRNA
PguCas13b_crRNA
MbCas12a_crRNA
SaCas9_gRNA
Trigger
LbCas12a
AkCas12b
AsCas12a
8.5x
6.9x
2.7x
9.2x
LbCas12a_crRNA
AkCas12b_crRNA 1
AkCas12b_crRNA 2
AsCas12a_crRNA
FnCas9
NcCas9
CasRx
1x
6.8x
7.9x
5.9x
7.3x
FnCas9_gRNA 1
FnCas9_gRNA 2
NcCas9_gRNA 1
NcCas9_gRNA 2
CasRx_crRNA

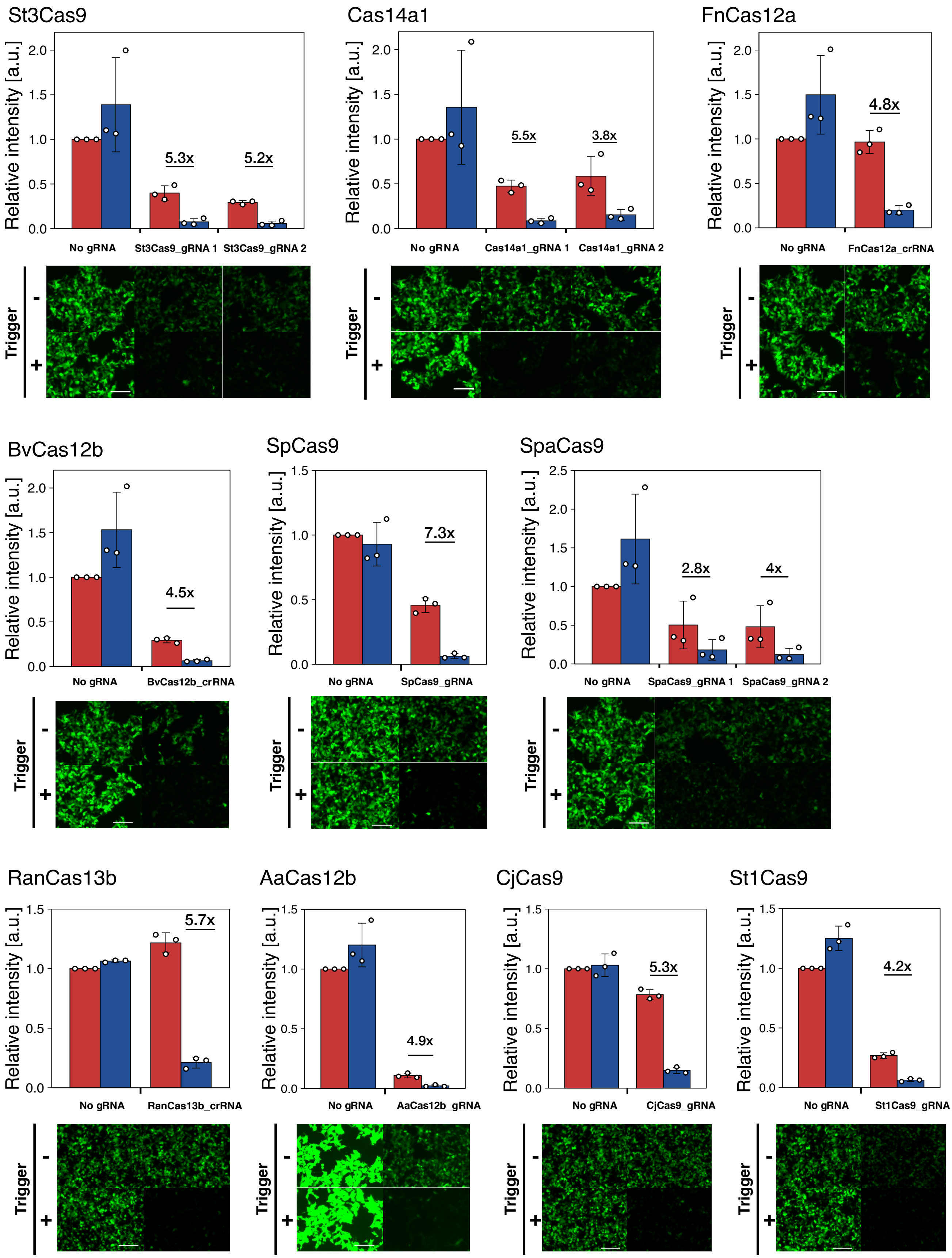
St3Cas9
Relative intensity [a.u.]
5.3x
5.2x
No gRNA
St3Cas9_gRNA 1
St3Cas9_gRNA 2
Trigger
-
+
Cas14a1
Relative intensity [a.u.]
5.5x
3.8x
No gRNA
Cas14a1_gRNA 1
Cas14a1_gRNA 2
Trigger
FnCas12a
Relative intensity [a.u.]
4.8x
No gRNA
FnCas12a_crRNA
Trigger
BvCas12b
Relative intensity [a.u.]
4.5x
No gRNA
BvCas12b_crRNA
Trigger
SpCas9
Relative intensity [a.u.]
7.3x
No gRNA
SpCas9_gRNA
Trigger
SpaCas9
Relative intensity [a.u.]
2.8x
4x
No gRNA
SpaCas9_gRNA 1
SpaCas9_gRNA 2
Trigger
RanCas13b
Relative intensity [a.u.]
5.7x
No gRNA
RanCas13b_crRNA
Trigger
AaCas12b
Relative intensity [a.u.]
4.9x
No gRNA
AaCas12b_gRNA
Trigger
CjCas9
Relative intensity [a.u.]
5.3x
No gRNA
CjCas9_gRNA
Trigger
St1Cas9
Relative intensity [a.u.]
4.2x
No gRNA
St1Cas9_gRNA
Trigger

PlmCasX

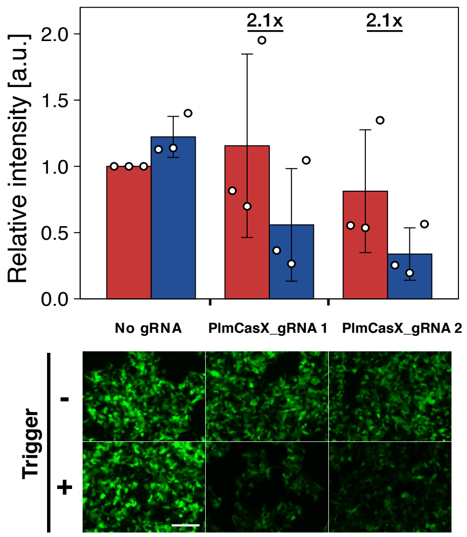

NmCas9

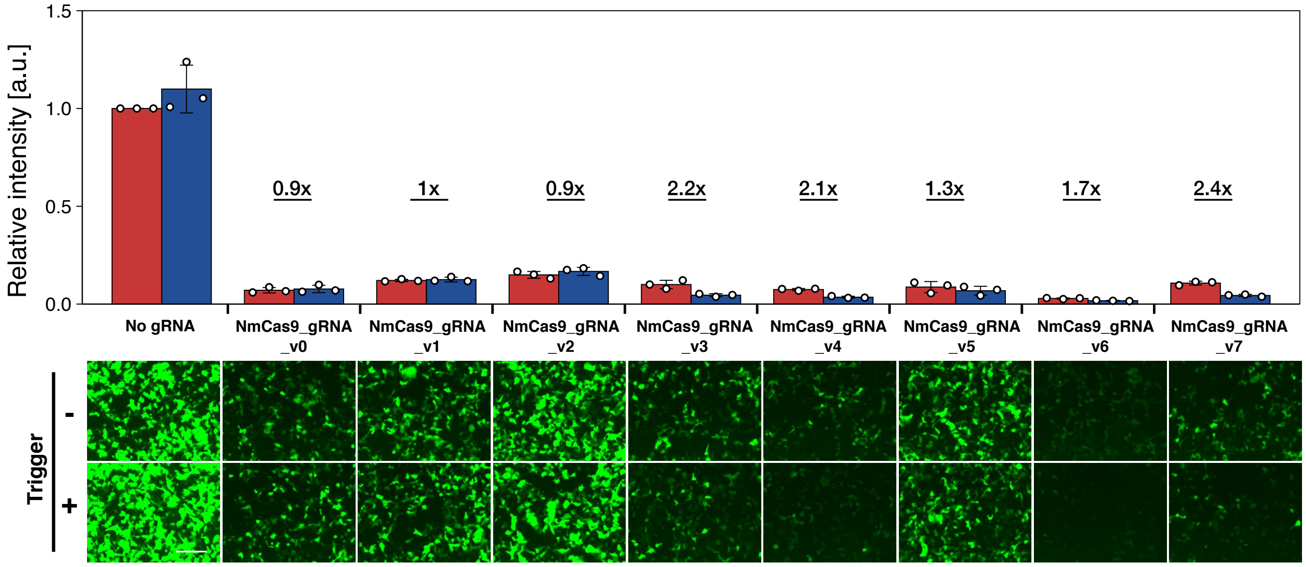

ClCas9

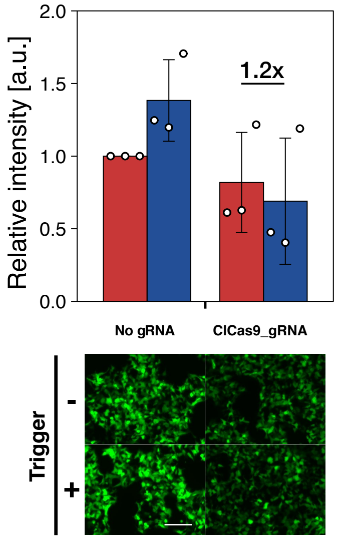

PlCas9

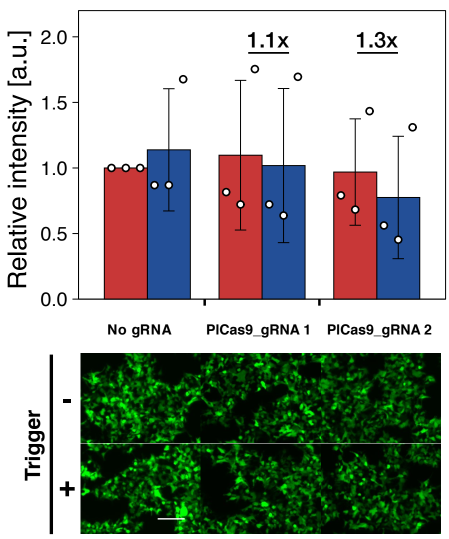

CdCas9

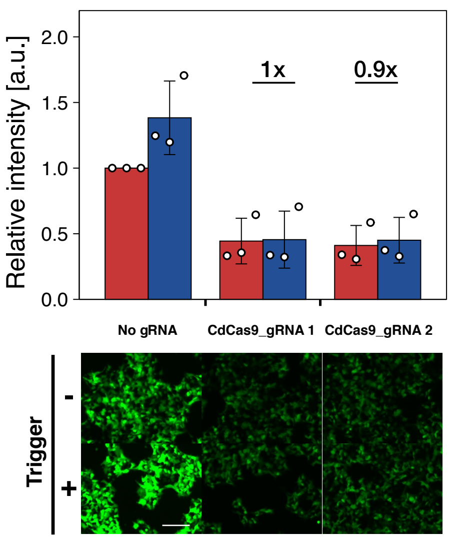

Supplementary Figure 2|
Original validation data of each Cas-responsive switch related to Figure 1E.
Validated trigger Cas proteins are stated at the top. The bar charts show the calculated reporter expression levels. HEK293FT cells were used in these experiments. Data are represented as the mean ± SD from three independent experiments. ON (trigger -) and OFF (trigger +) states are indicated in red and blue, respectively. Scale bars in cell images represent 200 μm. a.u.: arbitrary unit. Source data are provided as a Source Data file.

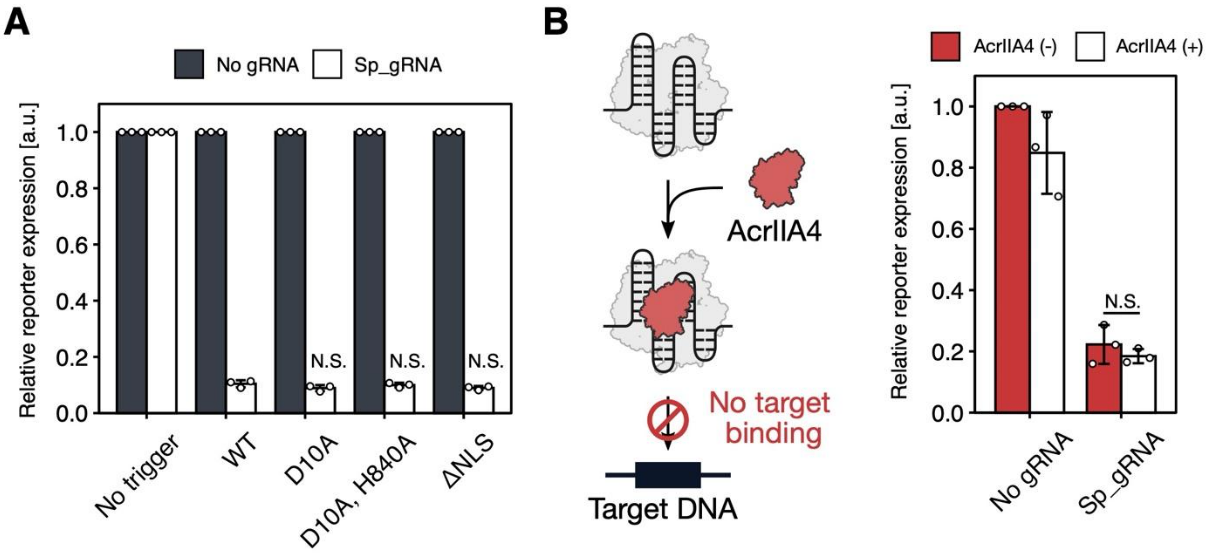

Supplementary Figure 3|
SpCas9-responsive reporter repression is independent of transcriptional regulations.
(A) Relative reporter expression levels in SpCas9 mutant transfections. Each SpCas9 mutant did not affect the relative reporter expression levels of Sp_gRNA compared with wild type (WT). (B) Schematic diagram depicting AcrIIA4-mediated binding inhibition between DNA and SpCas9 RNP complex (left). Relative reporter expression levels in co-transfections with AcrIIA4 (right). HEK293FT cells were used in these experiments. Data are represented as the mean ± SD from three independent experiments. Statistical analyses were performed using the unpaired two-tailed Student's *t*-test. N.S., not significant ($p \geq 0.05$). a.u.: arbitrary unit. Source data are provided as a Source Data file.

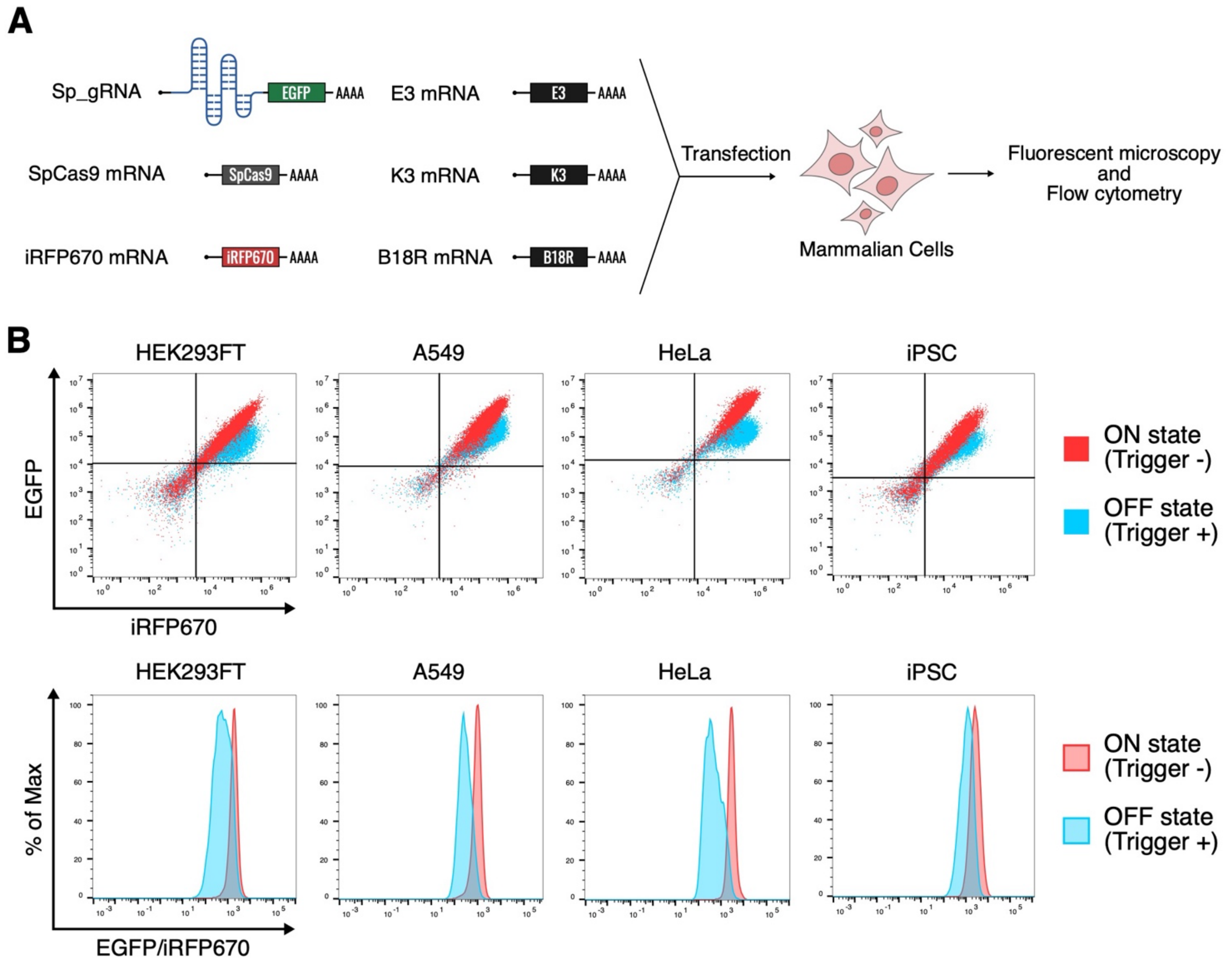

Supplementary Figure 4|

The performance of SpCas9-responsive switch in RNA transfection.

(A) Schematic diagram of experimental procedures in RNA transfection. Three mRNAs (switch and trigger mRNA (SpCas9 mRNA), and reference mRNA (iRFP670 mRNA)) were transfected into HEK293FT and iPS cells. In the case of HeLa and A549 cells, E3, K3, and B18R mRNAs were transfected in addition to the above three mRNAs to reduce immune response. The cells were analyzed by fluorescence microscopy and flow cytometer. (B) Representative dot plots and histograms of expression levels from switch mRNA with (blue) and without (red) SpCas9 mRNA. Three independent experiments were performed with similar results.

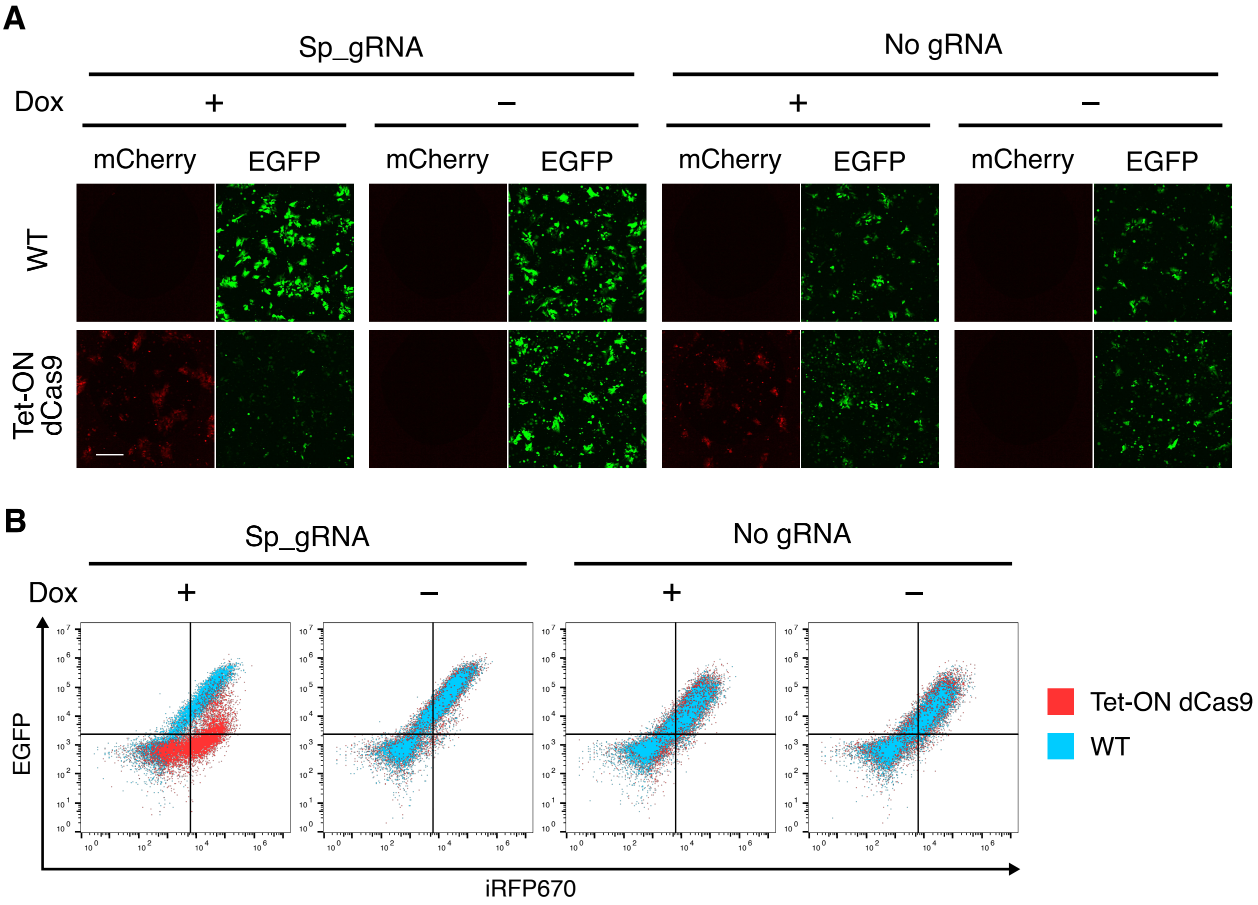

Supplementary Figure 5|

The performance of SpCas9-responsive switch for the iPSC genome integrated Cas9.

(A) Representative fluorescence microscopy images of each condition. Scale bar, 200 μm. Three independent experiments were performed with similar results. (B) Representative dot plots of each condition. Red and blue dots indicate Tet-ON dCas9 iPSCs and WT iPSCs, respectively. The x- and y-axes show EGFP and iRFP670, respectively. Efficient EGFP repression was observed when dSpCas9 expression was induced by Dox treatment.

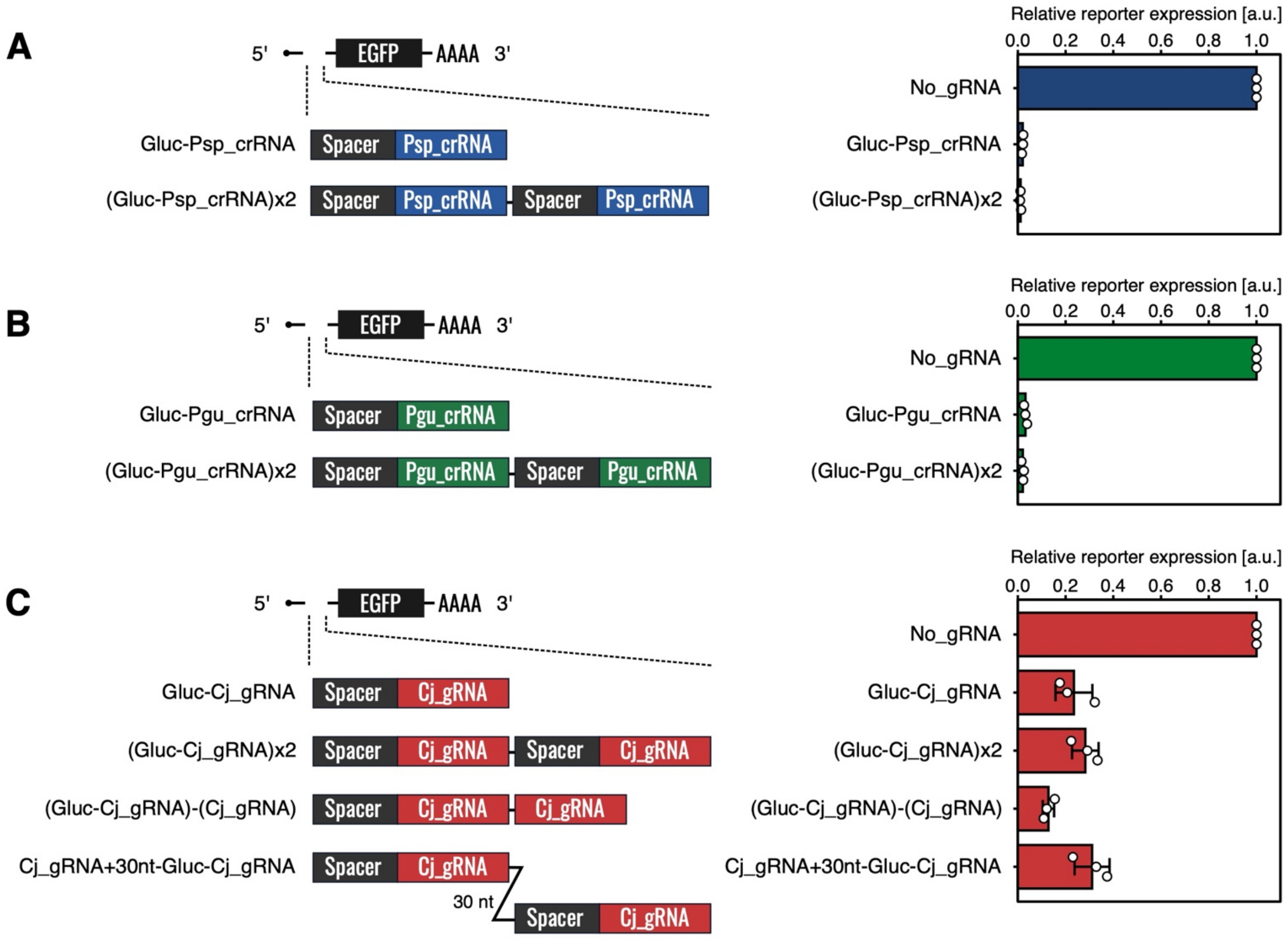

Supplementary Figure 6|
The effects of tandem insertion of sgRNAs.
(A-C) Schematic diagram depicting inserted sgRNAs, and relative reporter expression levels of each switch. (A) PspCas13b-responsive switch. (B) PguCas13b-responsive switch. (C) CjCas9-responsive switch. HEK293FT cells were used in these experiments. Data are represented as the mean ± SD from three independent experiments. a.u.: arbitrary unit. Source data are provided as a Source Data file.

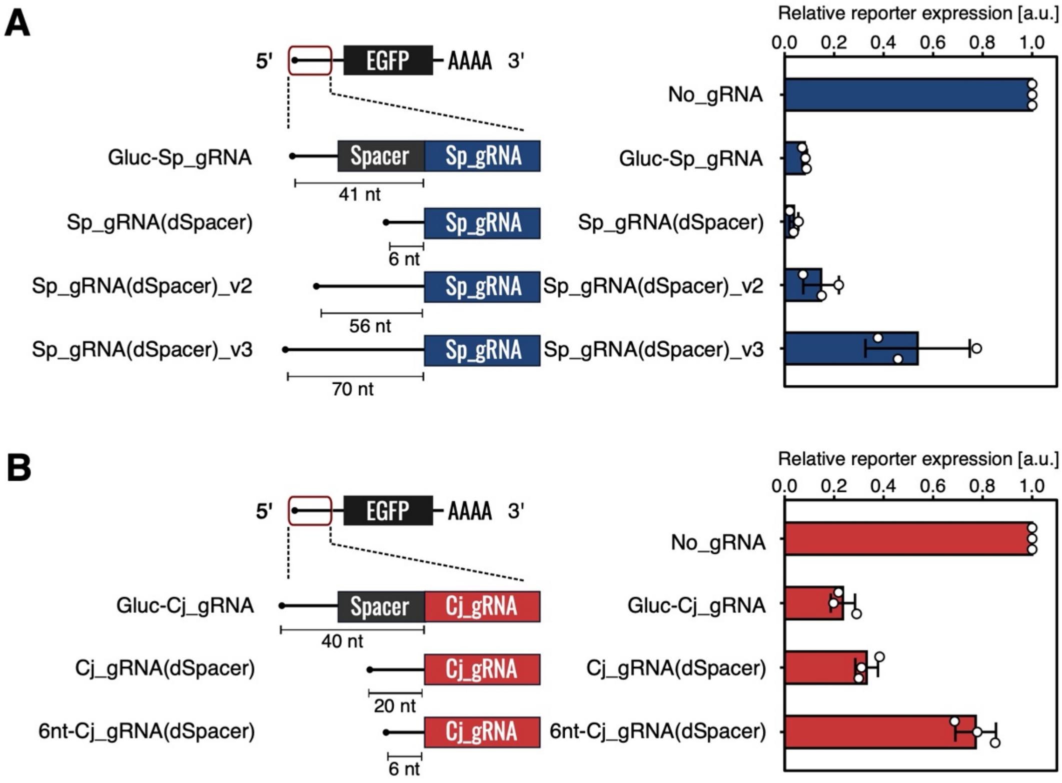

Supplementary Figure 7|
The validation of the position of sgRNA insertion.
(A, B) Schematic diagram depicting inserted sgRNAs, and relative reporter expression levels of each switch. (A) SpCas9-responsive switch. (B) CjCas9-responsive switch. HEK293FT cells were used in these experiments. Data are represented as the mean ± SD from three independent experiments. a.u.: arbitrary unit. Source data are provided as a Source Data file.

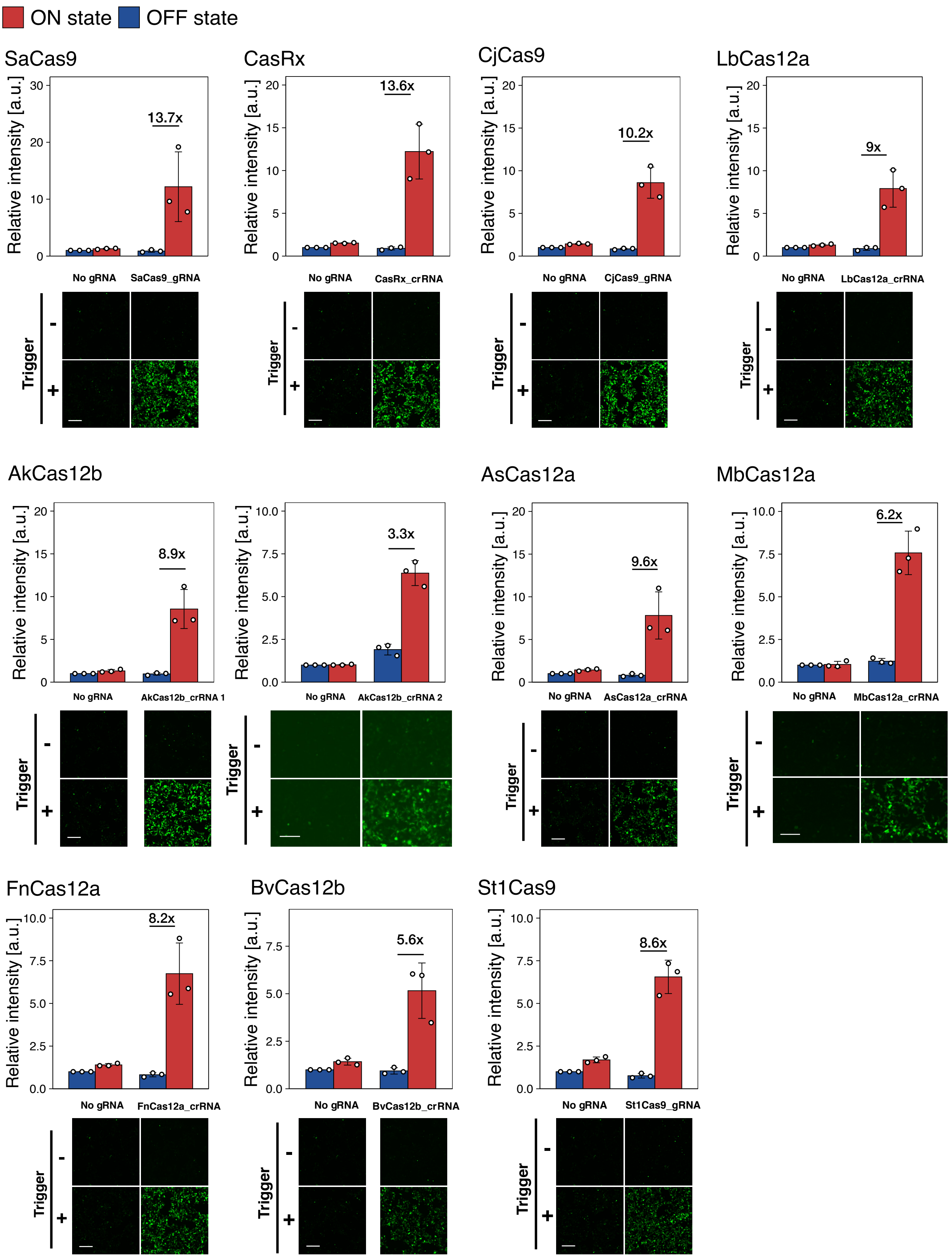

ON state
OFF state
SaCas9
Relative intensity [a.u.]
13.7x
No gRNA
SaCas9_gRNA
CasRx
13.6x
CasRx_crRNA
CjCas9
10.2x
CjCas9_gRNA
LbCas12a
9x
LbCas12a_crRNA
AkCas12b
8.9x
AkCas12b_crRNA 1
3.3x
AkCas12b_crRNA 2
AsCas12a
9.6x
AsCas12a_crRNA
MbCas12a
6.2x
MbCas12a_crRNA
FnCas12a
8.2x
FnCas12a_crRNA
BvCas12b
5.6x
BvCas12b_crRNA
St1Cas9
8.6x
St1Cas9_gRNA
Trigger
-
+

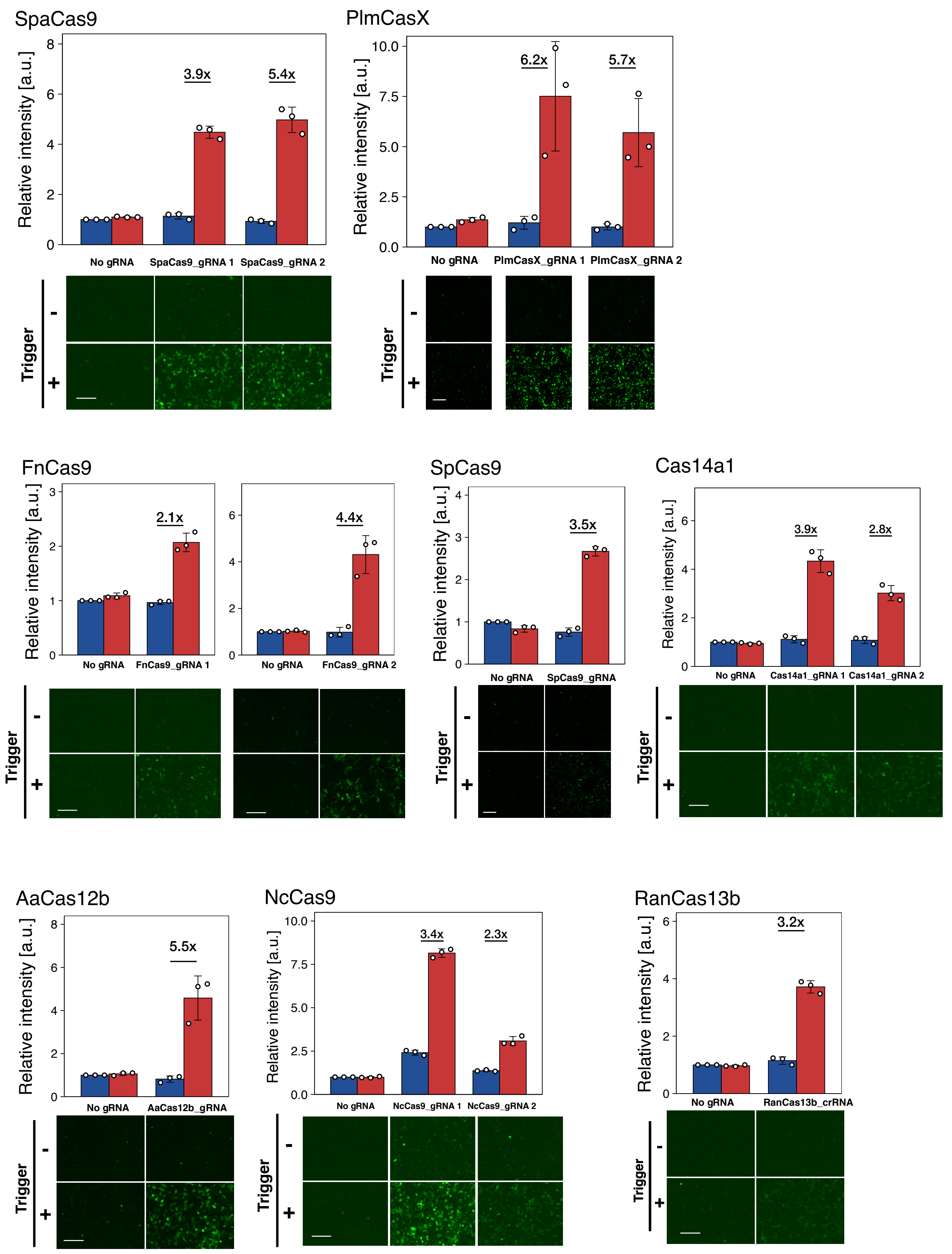
SpaCas9
Relative intensity [a.u.]
3.9x
5.4x
No gRNA
SpaCas9_gRNA 1
SpaCas9_gRNA 2
Trigger
PlmCasX
6.2x
5.7x
PlmCasX_gRNA 1
PlmCasX_gRNA 2
FnCas9
2.1x
4.4x
FnCas9_gRNA 1
FnCas9_gRNA 2
SpCas9
3.5x
SpCas9_gRNA
Cas14a1
3.9x
2.8x
Cas14a1_gRNA 1
Cas14a1_gRNA 2
AaCas12b
5.5x
AaCas12b_gRNA
NcCas9
3.4x
2.3x
NcCas9_gRNA 1
NcCas9_gRNA 2
RanCas13b
3.2x
RanCas13b_crRNA

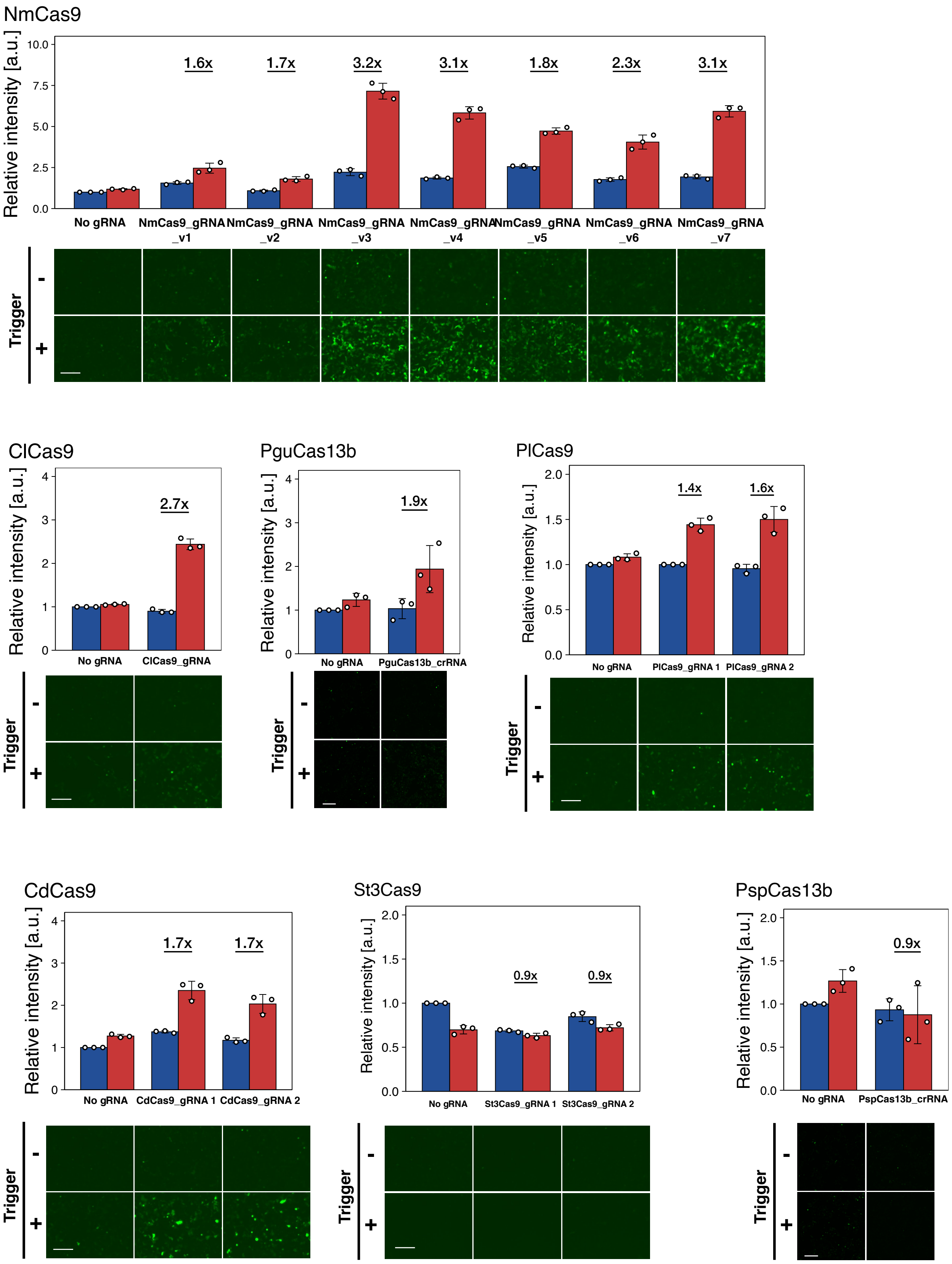

Supplementary Figure 8|
Original validation data of each Cas-responsive ON switch related to Figure 2B.
Validated trigger Cas proteins are stated at the top. Bar charts show the calculated reporter expression levels. Error bars represent standard deviations. OFF (trigger -) and ON (trigger +) states are indicated in blue and red, respectively. HEK293FT cells were used in these experiments. Scale bars in cell images represent 200 μm. a.u.: arbitrary unit. Source data are provided as a Source Data file.

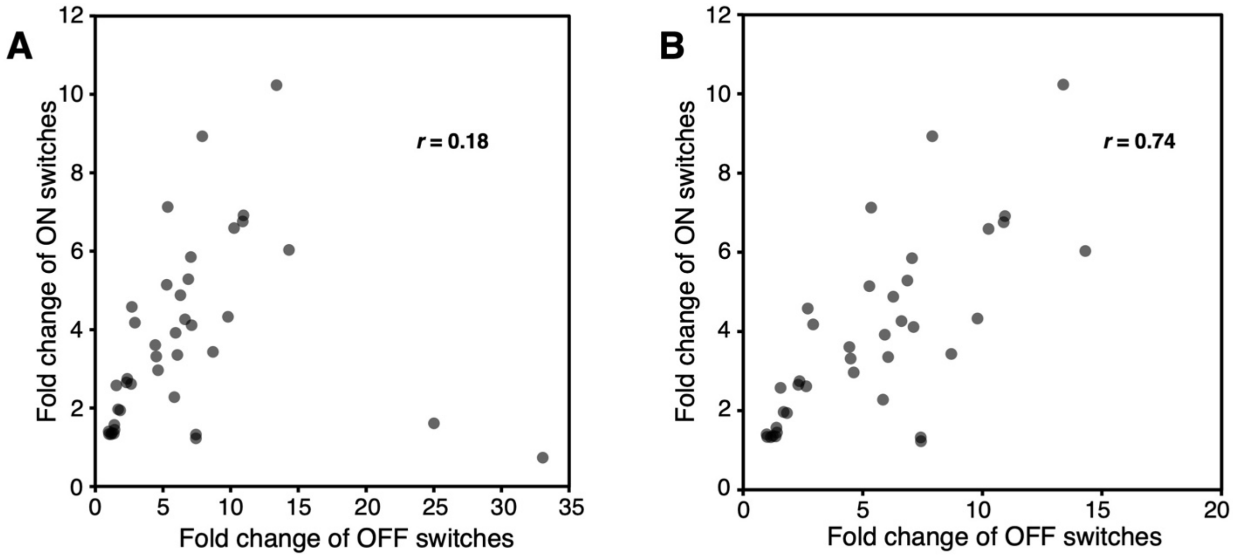

Supplementary Figure 9|

Correlation of the performance in Cas-responsive OFF switches and ON switches.

(A) Comparison of all Cas-responsive OFF and ON switches shown in Figures 1E and Figure 2B. Fold Change of OFF switch was calculated as the inverse of the relative reporter expression levels. (B) Comparison of Cas-responsive OFF and ON switches without PspCas13b- and PguCas13b-responsive ones. Pearson correlation coefficients (*r*) are shown in the charts. Source data are provided as a Source Data file.

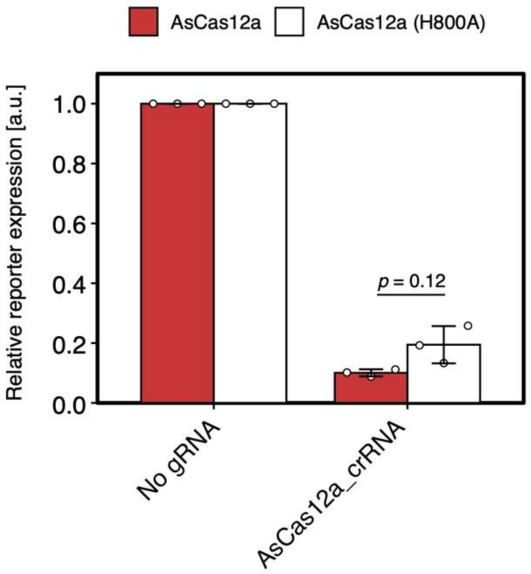

Supplementary Figure 10|
Effects of RNase activity on the AsCas12a-responsive switch.
Comparison of translational repression efficiency between wild type AsCas12a and its mutant (H800A). AsCas12a (H800A) lacks RNase activity but maintains other enzymatic activities. Values were normalized by the value in the condition when WT and "No gRNA" reporter plasmids were co-transfected. Data are presented as the mean ± SD from three independent experiments with HEK293FT cells. Error bars represent standard deviations. Statistical analyses were performed by using the unpaired two-tailed Student's *t*-test. a.u.: arbitrary unit. Source data are provided as a Source Data file.

A

Pearson (*r*)	ON state expression level (a.u.)	Repression efficiency (%)	MFE of Inserted RNA (kcal/mol)	MFE of Whole 5'UTR (kcal/mol)
OFF state expression level (a.u.)	0.475	-0.513	0.028	-0.009
ON state expression level (a.u.)		**0.242**	0.679	**0.655**
Repression efficiency (%)			0.393	**0.410**
MFE of Inserted RNA (kcal/mol)				0.991

B

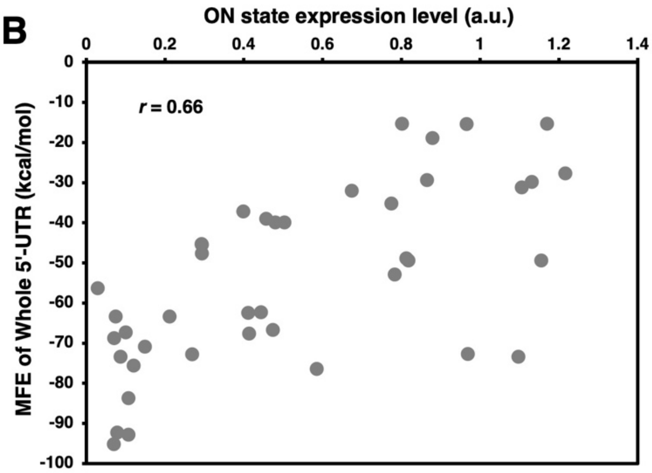

C

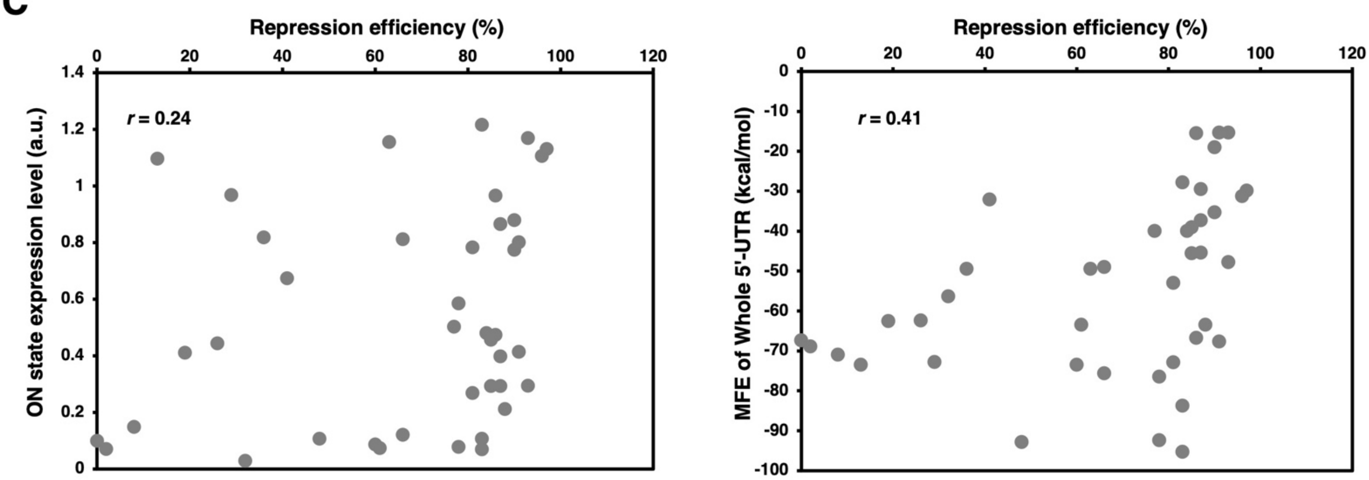

Supplementary Figure 11|
Relationships in verified parameters.
(A) Summary of Pearson correlation coefficients between comparisons of OFF state expression levels, ON state expression levels, repression efficiencies, MFEs of inserted RNA, and MFEs of whole 5'-UTR. The bold values are corresponding to Supplementary Figures 11B and C. (B, C) The two-dimension plots of (B) MFEs of whole 5'-UTR and ON state expression levels, (C) repression efficiencies and ON state expression levels (left), and MFEs of whole 5'-UTR and repression efficiencies (right). a.u.: arbitrary unit. Source data are provided as a Source Data file.

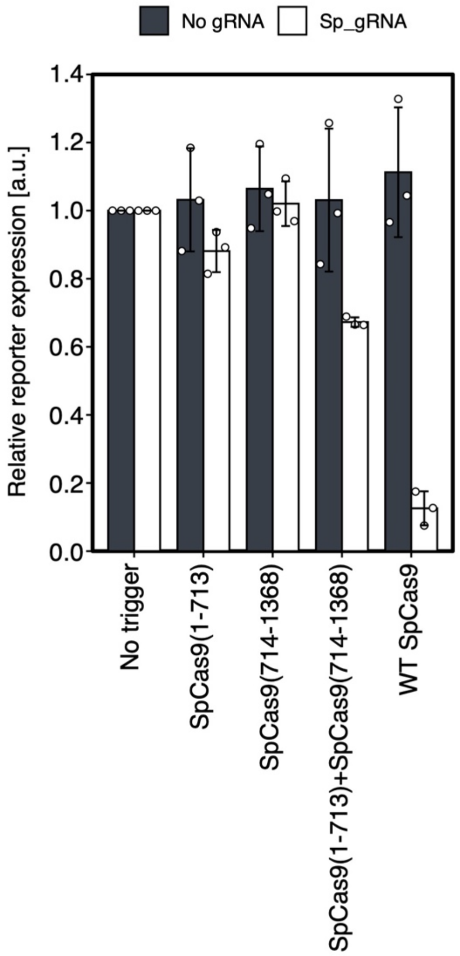

Supplementary Figure 12|
The translational repression with auto-assemble split-Cas9.
Translational repression occurred only when both protein fragments were present. The reporter assay showed a 33% repression when both input proteins were expressed. Data are presented as the mean ± SD from three independent experiments. HEK293FT cells were used in this experiment. a.u.: arbitrary unit. Source data are provided as a Source Data file.

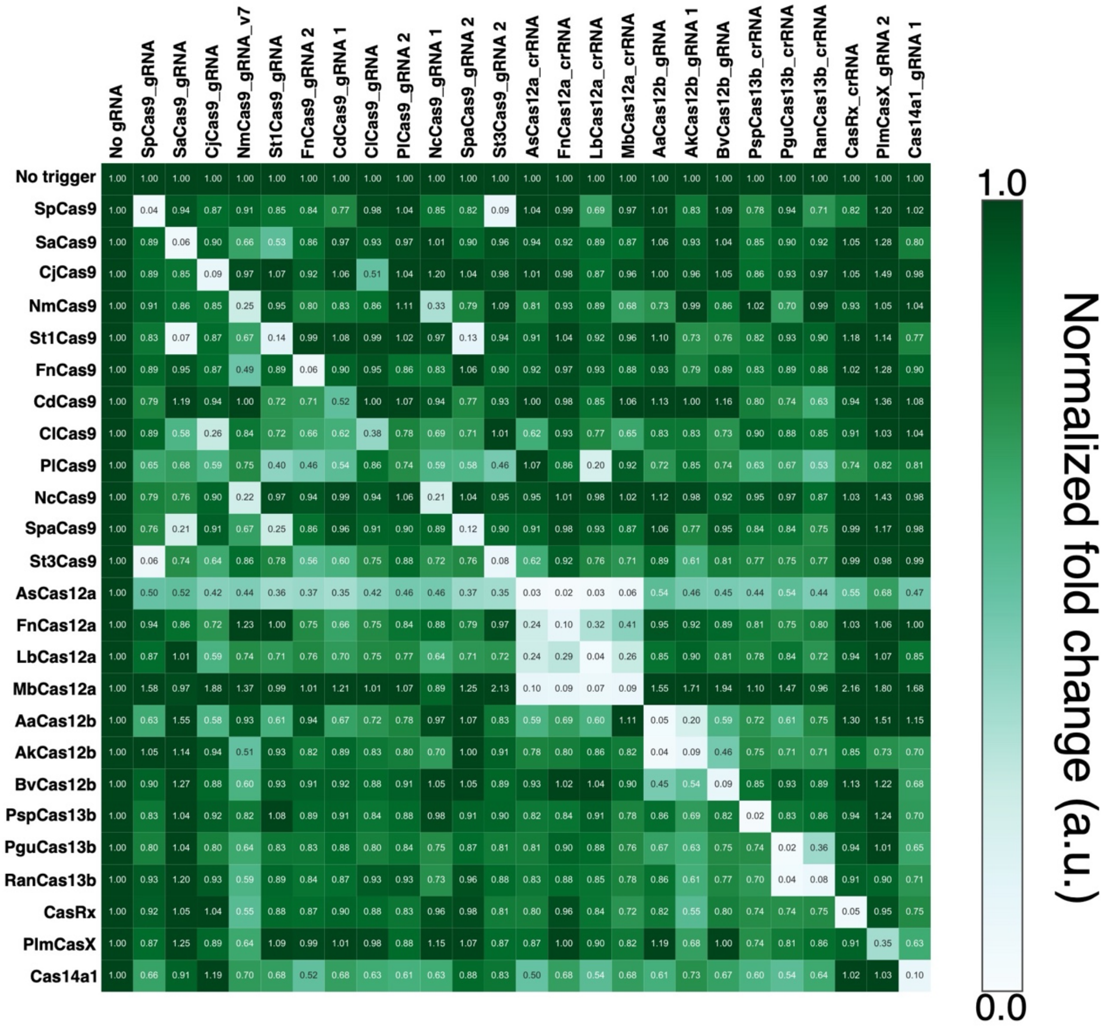

Supplementary Figure 13|

Orthogonality heat-map among representative Cas-responsive OFF switches related to Figure 4A.

Heat-map of the 25 × 25 orthogonality matrix representing Cas proteins and Cas-responsive switches with the indicated mean values from imaging analysis. Values indicate the mean calculated from imaging analysis of three independent experiments performed on different days. a.u.: arbitrary unit. Source data are provided as a Source Data file.

A

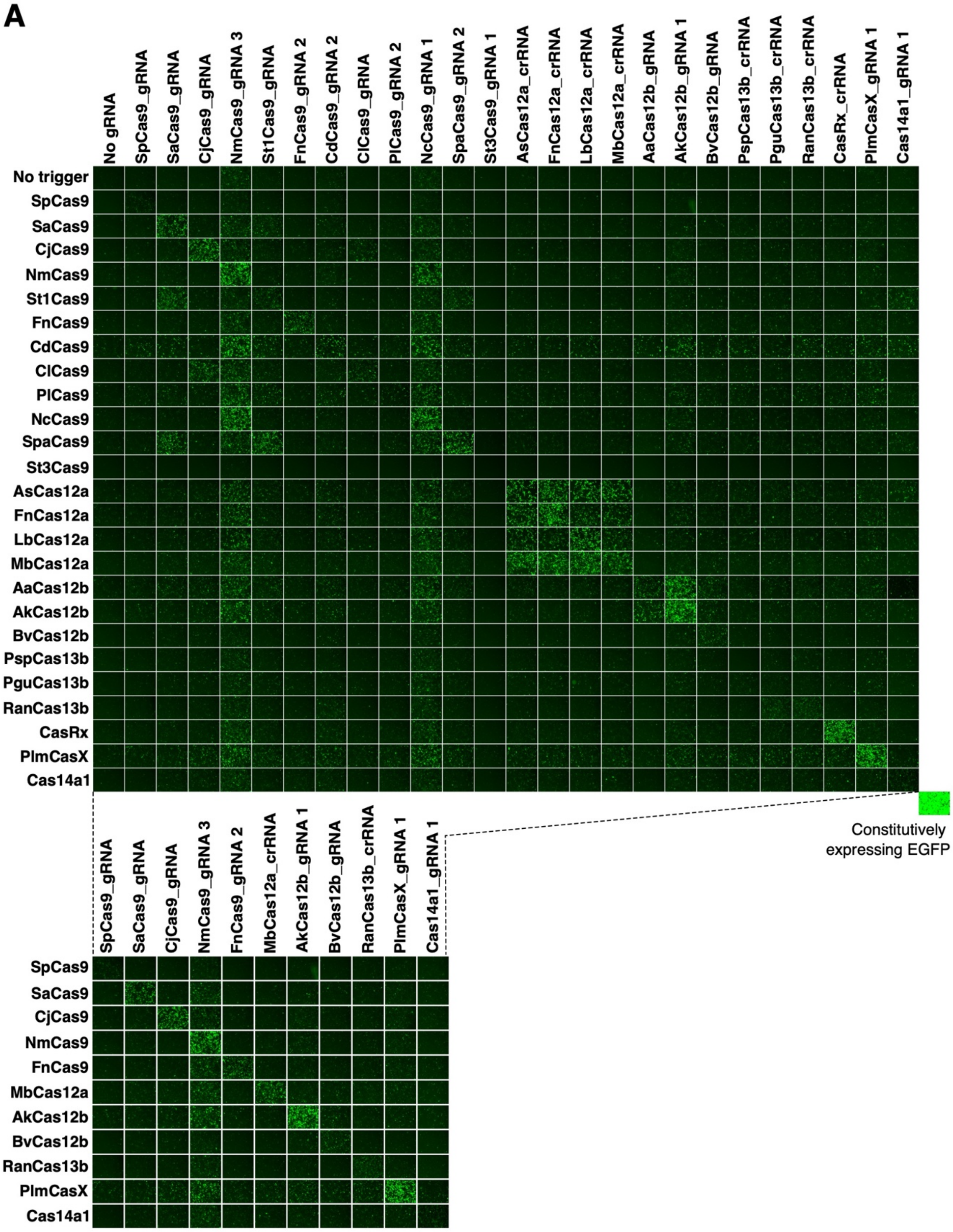
No gRNA
SpCas9_gRNA
SaCas9_gRNA
CjCas9_gRNA
NmCas9_gRNA 3
St1Cas9_gRNA
FnCas9_gRNA 2
CdCas9_gRNA 2
ClCas9_gRNA
PlCas9_gRNA 2
NcCas9_gRNA 1
SpaCas9_gRNA 2
St3Cas9_gRNA 1
AsCas12a_crRNA
FnCas12a_crRNA
LbCas12a_crRNA
MbCas12a_crRNA
AaCas12b_gRNA
AkCas12b_gRNA 1
BvCas12b_gRNA
PspCas13b_crRNA
PguCas13b_crRNA
RanCas13b_crRNA
CasRx_crRNA
PlmCasX_gRNA 1
Cas14a1_gRNA 1
No trigger
SpCas9
SaCas9
CjCas9
NmCas9
St1Cas9
FnCas9
CdCas9
ClCas9
PlCas9
NcCas9
SpaCas9
St3Cas9
AsCas12a
FnCas12a
LbCas12a
MbCas12a
AaCas12b
AkCas12b
BvCas12b
PspCas13b
PguCas13b
RanCas13b
CasRx
PlmCasX
Cas14a1
Constitutively expressing EGFP
SpCas9_gRNA
SaCas9_gRNA
CjCas9_gRNA
NmCas9_gRNA 3
FnCas9_gRNA 2
MbCas12a_crRNA
AkCas12b_gRNA 1
BvCas12b_gRNA
RanCas13b_crRNA
PlmCasX_gRNA 1
Cas14a1_gRNA 1
SpCas9
SaCas9
CjCas9
NmCas9
FnCas9
MbCas12a
AkCas12b
BvCas12b
RanCas13b
PlmCasX
Cas14a1

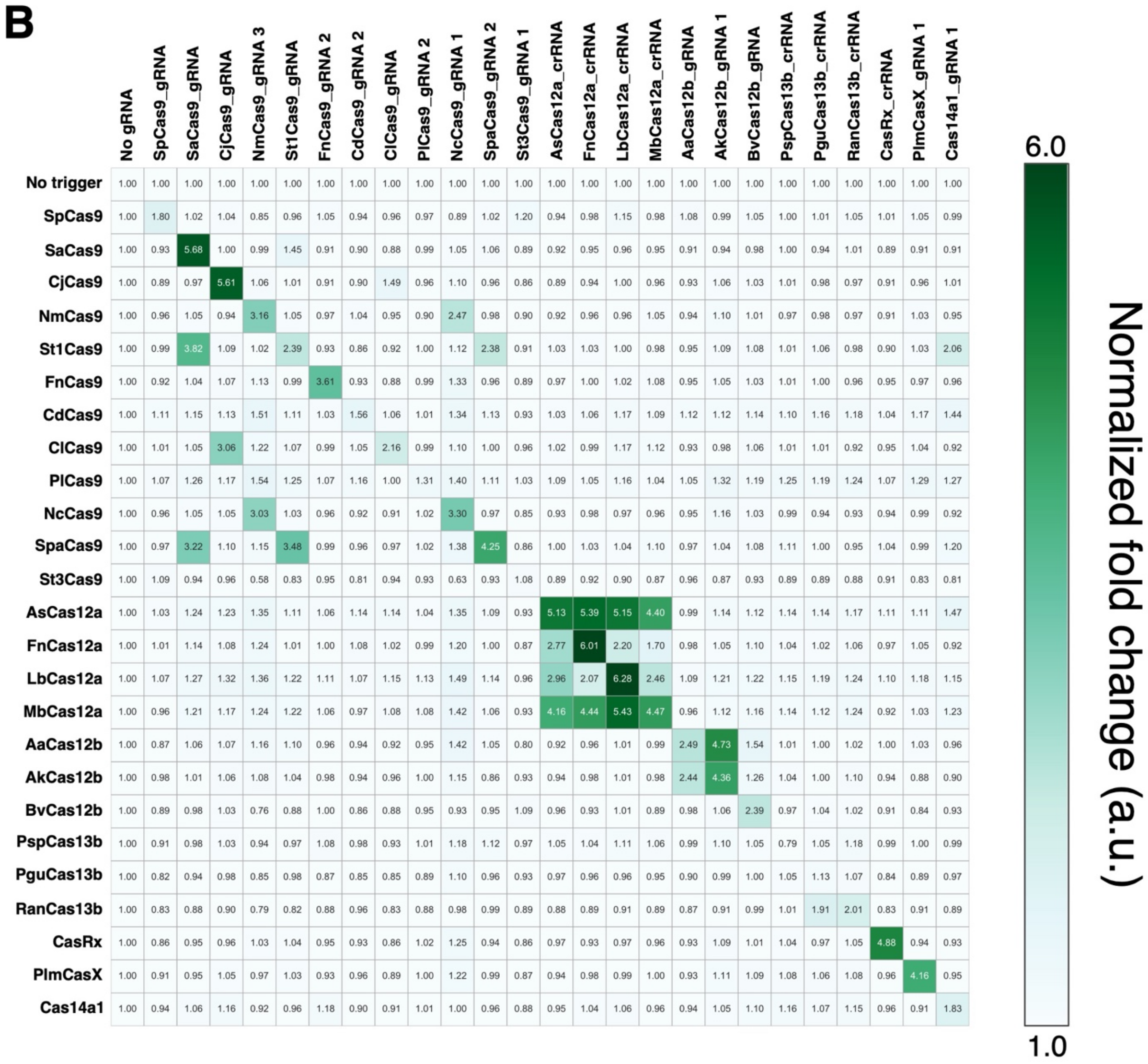

Supplementary Figure 14|

Fluorescence cell images and heat map of the 25 × 25 orthogonality matrix of representative Cas proteins and Cas-responsive ON switches.

(**A**) A fluorescence image of cells transfected with a plasmid constitutively expressing EGFP is shown at the bottom right corner of the upper panel for reference. Pruned images at the bottom indicate mutually orthogonal sets. **(B)** Values indicate the mean calculated from imaging analysis of three independent experiments performed on different days. HEK293FT cells were used in these experiments. a.u.: arbitrary unit. Source data are provided as a Source Data file.

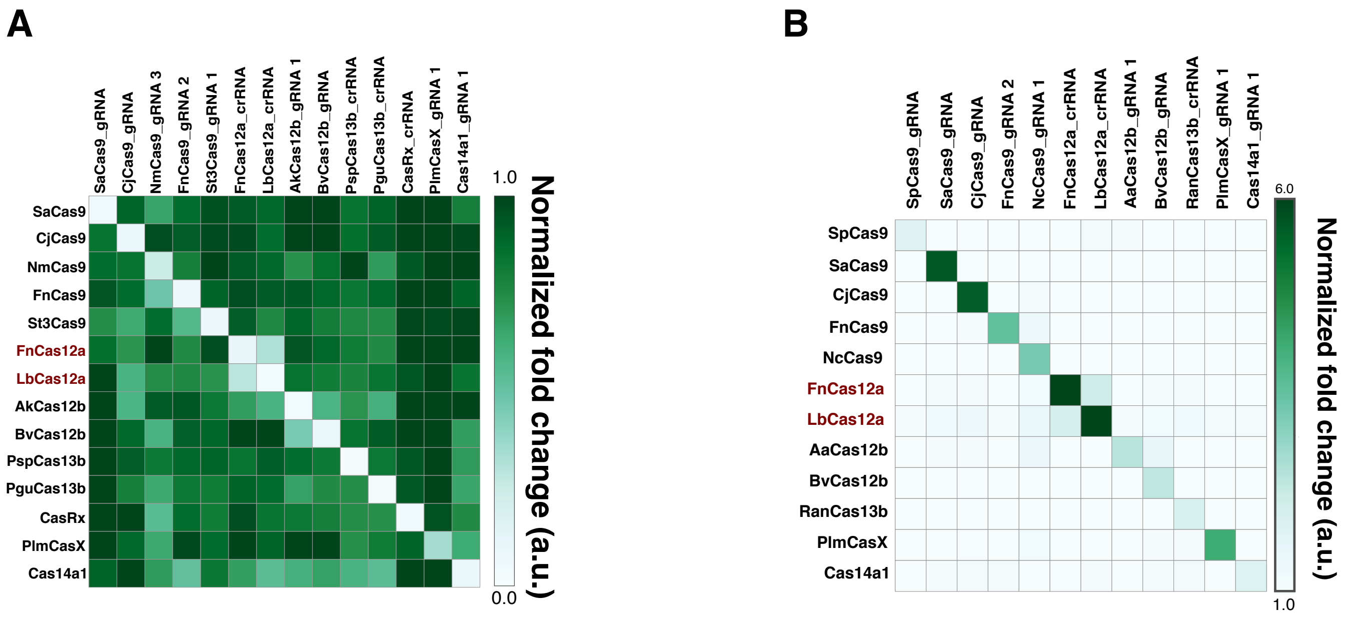

Supplementary Figure 15|

Extraction of orthogonal pairs in Cas proteins and Cas-responsive OFF and ON switches related to Figure 4.

(**A**) Combinations which are lower than the threshold of 0.7 as OFF switches. Because the set between FnCas12a and LbCas12a-responsive switches, which showed obvious crosstalk, was selected as the best combination, we concluded that there were no 14-Cas proteins combinations that met our criteria for orthogonality. (**B**) Combinations which were lower than the threshold of 0.7 as ON switches. FnCas12a and LbCas12a-responsive switches were selected as the best combination in the set which selected 12 Cas proteins (right), whereas the set with 11 Cas proteins did not contain them (see Figure 4D). a.u.: arbitrary unit. Source data are provided as a Source Data file.

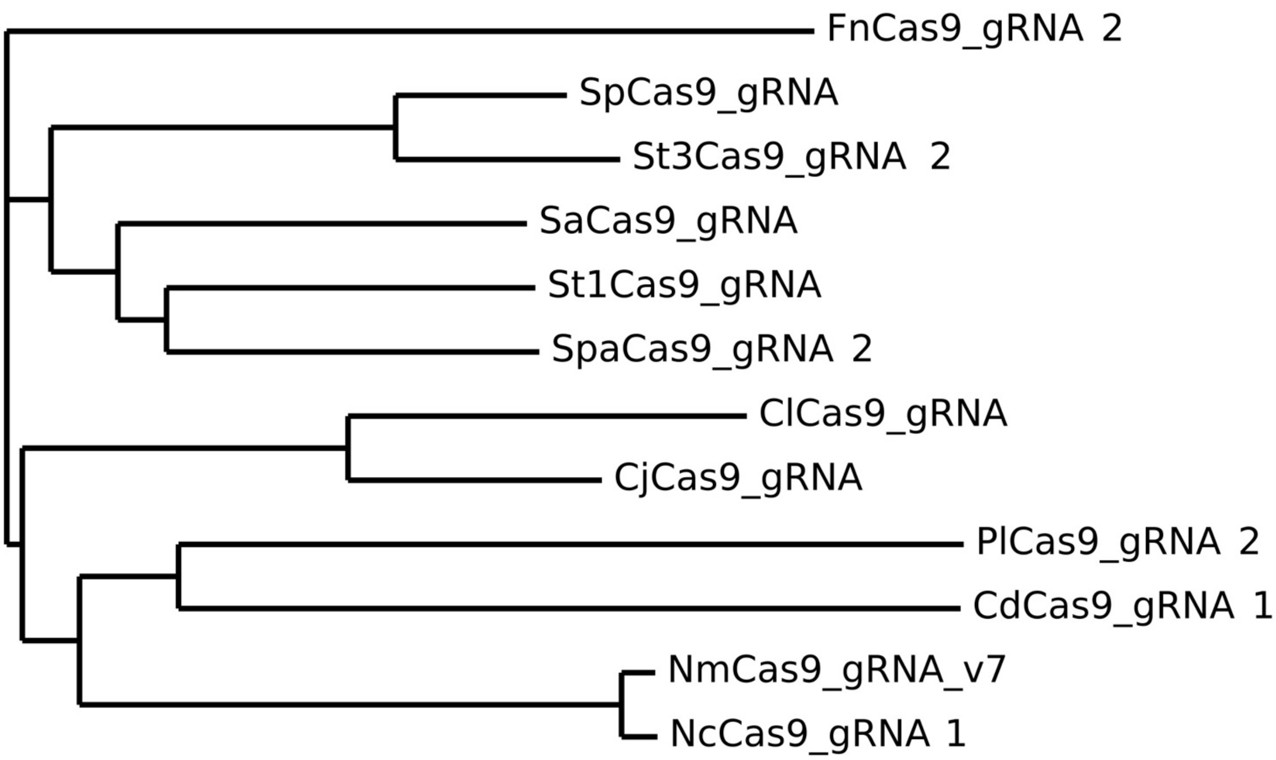

Supplementary Figure 16|
Phylogenetic tree of Cas9 sgRNA used in OFF switch orthogonality.
Sequences were aligned using the MUSCLE web tool. The pairs that showed crosstalk were grouped in closer clusters. Source data are provided as a Source Data file.

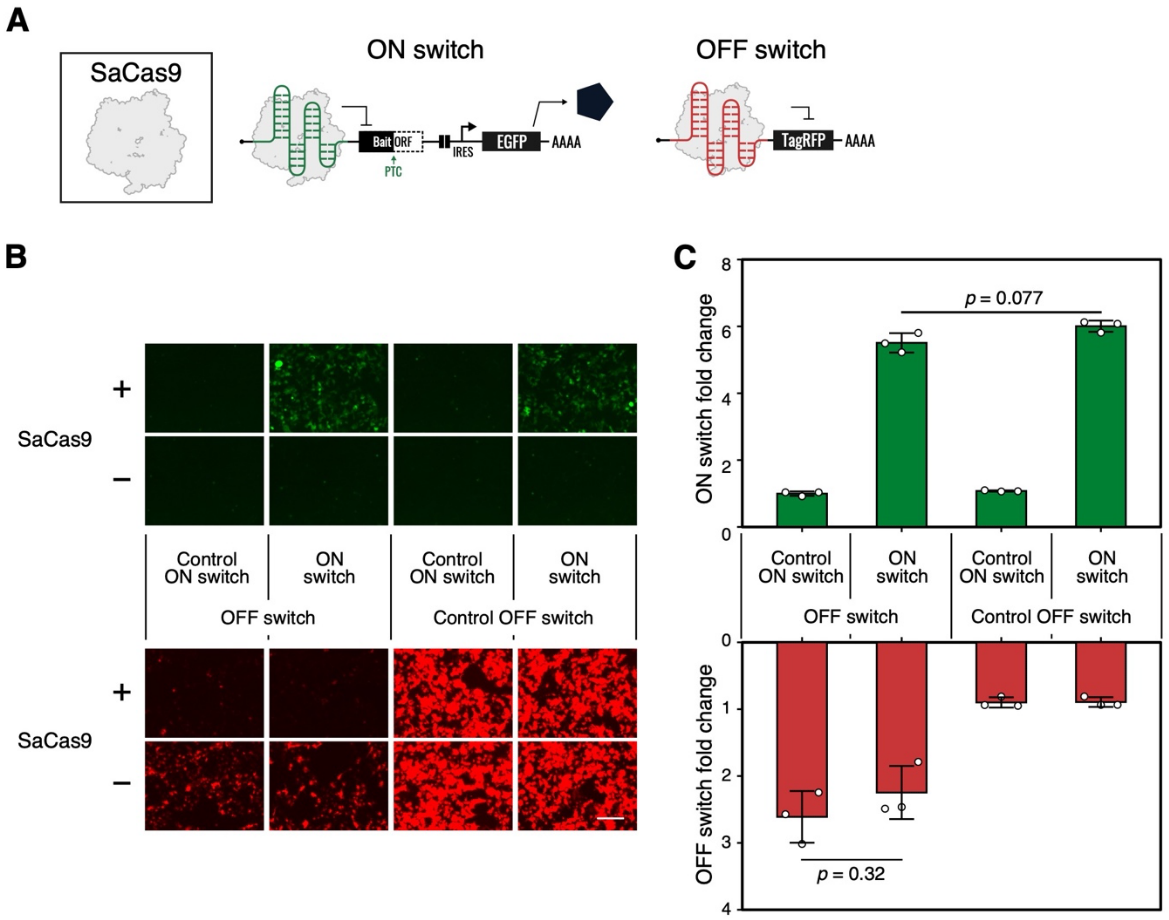

Supplementary Figure 17|

Simultaneous regulation of translational activation and repression with SaCas9.

(A) Schematic diagram of the simultaneous translational regulation system. SaCas9 was used as the trigger protein. Translational activation and repression were monitored by EGFP and TagRFP fluorescence, respectively. (B) Representative fluorescence microscopic images from each condition. Translational activation and repression were observed simultaneously when SaCas9 was transfected. Scale bar, 200 μm. (C) Quantitative data of reporter expression levels were obtained by flow cytometer. OFF switch: a plasmid for expressing TagRFP whose translation is repressed by SaCas9. ON switch: a plasmid for expressing EGFP whose translation is activated by SaCas9. Control OFF and ON switch: plasmids without the sgRNA sequence for SaCas9. HEK293FT cells were used in this experiment. Error bars represent mean ± SD from three independent experiments. Statistical analyses were performed by using the unpaired two-tailed Student's *t*-test. Source data are provided as a Source Data file.

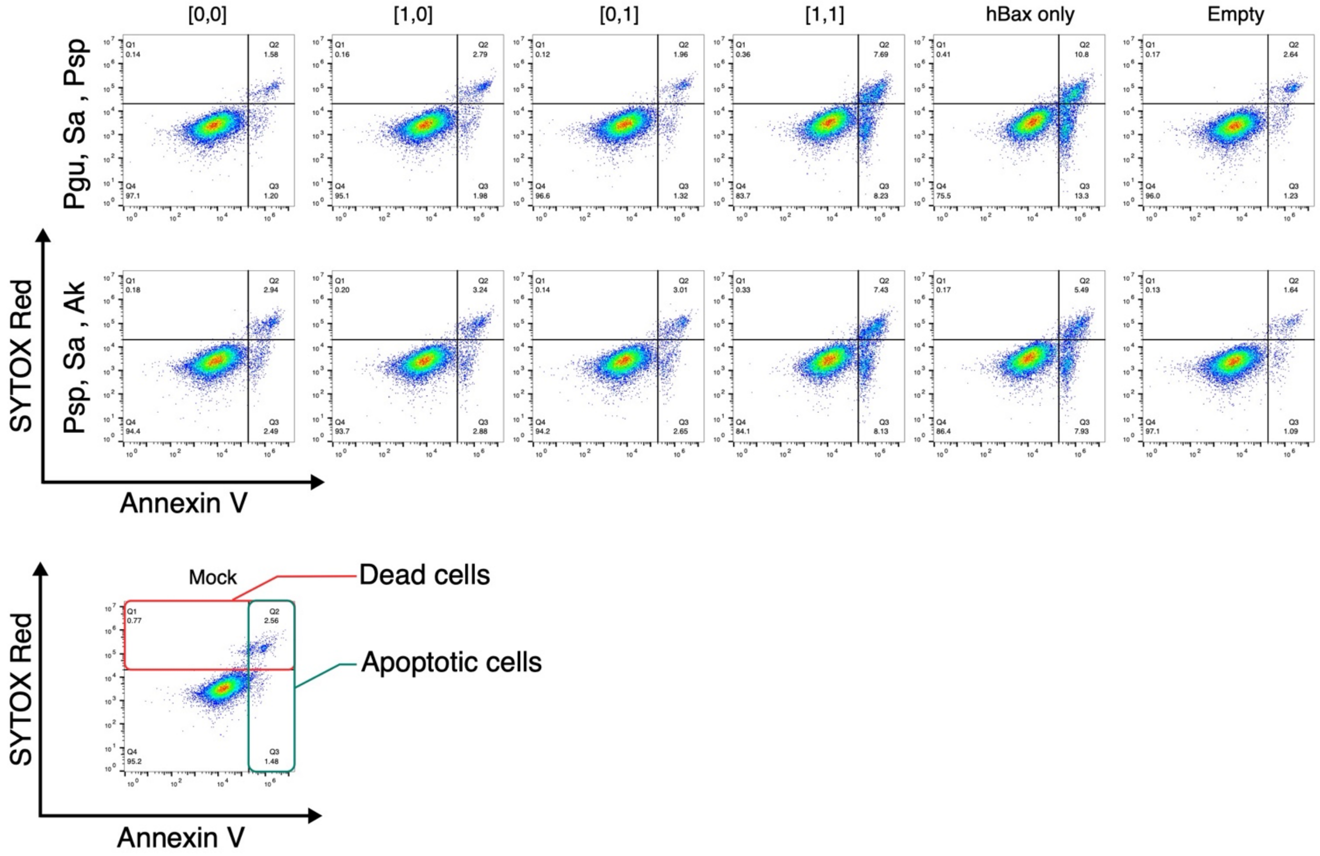

Supplementary Figure 18|
Cell phenotype control with AND gate circuits related to Figure 6E, F.
Representative dot plots of cell viability. The top indicates AND gate consisting of PguCas13b and SaCas9 as inputs and PspCas13b as a mediator. The bottom shows AND gate composed of PspCas13b and SaCas9 as inputs and AkCas12b as a mediator. The x- and y-axes indicate Annexin V and SYTOX Red, respectively. Q1 + Q2 and Q2 + Q3 indicate dead cells and apoptotic cells, respectively. Increases in apoptotic and dead cells were observed only when both inputs were introduced into HEK293FT cells with either AND gate, similar to when a constitutively active hBax plasmid was transfected into the cells.

Supplementary Text 1: Investigating the strategies for improving Cas-responsive switches.

We observed a weak response in our initial design of the NmCas9-responsive switch (NmCas9_gRNA v0), although this Cas protein has genome editing activity in mammalian cells[1], suggesting that the weak response was not due to inactivity in mammalian cells. We designed seven different types of NmCas9-responsive switches (variants 1-7) by altering the sgRNA sequences and found that variant 7 moderately improved the switch performance (Supplementary Figure 2 and Supplementary Table 2). Similar effects were observed in FnCas9, NcCas9, SpaCas9, AkCas12b, and Cas14a1-responsive switches. Thus, it is possible to improve the repression efficiency of Cas-responsive switches by validating sgRNA sequences.

Switch performance could also be affected by the copy number or insertion position of RNA motifs[2]. We first examined the effects of changing the copy number of sgRNAs and the manner in which they were inserted. Tandem insertion of the sgRNA sequences improved the performance of the PspCas13b and PguCas13b-responsive switches (Supplementary Figure 6A, B), whereas it did not change the efficiency of the CjCas9-responsive switch (Supplementary Figure 6C). The use of CjCas9_gRNA in which the spacer sequence was deleted (dSpacer) improved repression efficiency. In contrast, when we connected two sgRNA sequences through an additional 30nt linker to avoid the steric hindrance between two CjCas9 proteins, the repression efficiency was similar to the original construct (Cj_gRNA+30nt-Gluc-Cj_gRNA and Gluc-Cj_gRNA, Supplementary Figure 6C).

Additionally, we assessed the positional effect of the Cas protein binding site (Supplementary Figure 7). Using the dSpacer sequence, in which the spacer sequence was deleted from the sgRNAs used, repression efficiency of the SpCas9-responsive switch varied according to the distance from the 5'-end (Supplementary Figure 7A). However, repression efficiency of the CjCas9-responsive switch became worse (Supplementary Figure 7B). The triple helix in the sgRNA[3] may make the design strategy more complicated in the case of the CjCas9-responsive switches. Further elucidations are required to establish a better strategy to improve translational switches based on Cas protein-sgRNA interactions.

Supplementary Table 1
Information of Cas proteins used in this study.

	Name	Origin	RNA-guided nuclease activity in mammalian cells	ORF source	Size (bp)[*1]	References
1	SpCas9	*Streptococcus pyogenes*	+	Addgene ID #41815 or #60599	4140	4,5
2	SaCas9	*Staphylococcus aureus*	+	Addgene ID #61591	3345	6
3	CjCas9	*Campylobacter jejuni*	+	Addgene ID #89752	3012	3,7
4	NmCas9	*Neisseria meningitidis*	+	Addgene ID #47867	3333	1,8,9
5	St1Cas9	*Streptococcus thermophilus*	+	Addgene ID #65775	3402	10
6	FnCas9	*Francisella novicida*	+ (with proxy-CRISPR)	Addgene ID #68705	4950	11,12
7	CdCas9	*Corynebacterium diphtheriae*	+	Addgene ID #68314	3318	6
8	ClCas9	*Campylobacter lari* CF89-12	-	Addgene ID #68336	3075	6
9	PlCas9	*Parvibaculum lavamentivorans*	-	Addgene ID #68333	3177	6
10	NcCas9	*Neisseria cinerea*	+ (with proxy-CRISPR)	Addgene ID #68331	3312	6,12
11	SpaCas9	*Streptococcus pasteurianus*	+	Addgene ID #68322	3456	6
12	St3Cas9	*Streptococcus thermophilus*	+	Addgene ID #68337	4260	6,13
	Name	Origin	RNA-guided nuclease activity in mammalian cells	ORF source	Size (bp)[*1]	References
1	AsCpf1 (AsCas12a)	*Acidaminococcus sp.* BV3L6	+	Gift from Hotta lab	4062	14
2	FnCpf1 (FnCas12a)	*Francisella novicida* U112	+	Addgene ID #69976	4038	14,15
3	LbCpf1 (LbCas12a)	*Lachnospiraceae bacterium* ND2006	+	Addgene ID #69988	3822	14
4	MbCpf1 (MbCas12a)	*Moraxella bovoculi* 237	+	Addgene ID #69986	4257	14
5	AaCas12b	*Alicyclobacillus acidiphilus*	+	Addgene ID #121961	3447	16
6	AkCas12b	*Alicyclobacillus kakegawensis*	+	Addgene ID #121946	3597	16,17
7	BvCas12b	*Bacillus sp.* NSP2.1	+	Addgene ID #122445	3522	17
	Name	Origin	RNA-guided nuclease activity in mammalian cells	ORF source	Size (bp)[*1]	References
1	PspCas13b	*Prevotella sp.*	+	Synthesized	3429	18
2	PguCas13b	*Porphyromonas gulae*	+	Addgene ID #103861	3657	18
3	RanCas13b	*Riemerella anatipestifer*	+	Addgene ID #103855	3417	18
4	CasRx (RfxCas13d)	*Ruminococcus flavefaciens XPD3002*	+	Addgene ID #109049	3003	19
	Name	Origin	RNA-guided nuclease activity in mammalian cells	ORF source	Size (bp)[*1]	References
1	PlmCasX	*Planctomycetes*	+	Synthesized	2937	20
2	Cas14a1	Uncultured archaeron	+	Addgene ID #112500	1590	21

*1 The length of the ORF used in this study (including stop codon)

Supplementary Table 2

Information about the inserted RNA sequences and their translational repression efficiencies.
The sgRNA and crRNA scaffold sequences referred to the references in Supplementary Table 1.
Red letters represent a protospacer sequence[18] which is complementary to an exogenous gene, *Gaussia* luciferase[*1]. The bold AUG sequence denotes the potential start codon.

	Name		Inserted RNA sequence	sgRNA length (nt)[*2]	Repression efficiency (%)
1	SpCas9	v1	GAGAUCAGGGCAAACAGAACUGUUUUAGAGCUAGAAAUAGCAAGUUAAAAUAAGGCUAGUCCGUUAUCAACUUGAAAAAGUGGCACCGAGUCGGUGC	97	85
2	SaCas9	v1	GAGAUCAGGGCAAACAGAACGUUUUAGUACUCUGGAAACAGAAUCUACUAAAACAAGGCAAA**AUG**CCGUGUUUAUCUCGUCAACUUGUUGGCGAGAU	97	93
3	CjCas9	v1	GAGAUCAGGGCAAACAGAACGUUUUAGUCCCUGAAAAGGGACUAAAAUAAAGAGUUUGCGGGACUCUGCGGGGUUACAAUCCCCUAAAACCGC	93	81
4	NmCas9	v0	GAGAUCAGGGCAAACAGAACGUUGUAGCUCCCUUUCUCGAAAGAGAACCGUUGCUACAAUAAGGCCGUCUGAAAAG**AUG**UGCCGCAACGCUCUGCCCCUUAAAGCUUCUGCUUUAAGGGGC	121	2
		v1	GAGAUCAGGGCAAACAGAACGUUGUAGCUCCCUUUCUCGAAAGAGAACCGUUGCUACAAUAAGGCCGUCUGAAAAG**AUG**UGCCGCAACGCUCUGCCCCUUAAAGCUUCUGCUUUAAGGGGC	121	8
		v2	GAGAUCAGGGCAAACAGAACGUUGUAGCUCCCUUUCUCGAAAGAGAACCGUUGCUACAAUAAGGCCGUCUGAAAAG**AUG**UGCCGCAACGCUCUGCCCCUUAAAGCUUCUGCUUUAAGGGGCAA	123	0
		v3	GUUGUAGCUCCCUUUCUCGAAAGAGAACCGUUGCUACAAUAAGGCCGUCUGAAAAG**AUG**UGCCGCAACGCUCUGCCCCUUAAAGCUUCUGCUUUAAGGGGCAUCGUUUA	109	61
		v4	GUUGUAGCUCCCUUUCUCAUUUCGGAAACGAA**AUG**AGAACCGUUGCUACAAUAAGGCCGUCUGAAAAG**AUG**UGCCGCAACGCUCUGCCCCUUAAAGCUUCUGCUUUAAGGGGCAUCGUUUA	121	60
		v5	GUUGUAGCUCCCUUUCGAACCGUUGCUACAAUAAGGCCGUCUGAAAAG**AUG**UGCCGCAACGCUCUGCCCCUUAAAGCUUCUGCUUUAAGGGGCAUCGUUUA	101	32
		v6	GUUGUAGCUCCCUUUCUCAUUUCGCAGUGCUACA**AUG**AAAAUUGUCGCACUGCGAA**AUG**AGAACCGUUGCUACAAUAAGGCCGUCUGAAAAG**AUG**UGCCGCAACGCUCUGCCCCUUAAAGCUUCUGCUUUAAGGGGCAUCGUUUA	145	48
		v7	GUUGUAGCUCCCUUUCUCUCCUGGAAACAGGAGAGAACCGUUGCUACAAUAAGGCCGUCUGAAAAG**AUG**UGCCGCAACGCUCUGCCCCUUAAAGCUUCUGCUUUAAGGGGCAUCGUUUA	119	66
5	St1Cas9	v1	GAGAUCAGGGCAAACAGAACGUUUUUGUACUCUCAAGAUUUAAGUAACUGUACAACGAAACUUACACAGUUACUUAAAUCUUGCAGAAGCUACAAAGAUAAGGCUUC**AUG**CCGAAAUCAACACCCUGUCAUUUU**AUG**GCAGGGUGUU	147	81
6	FnCas9	v1	GUUUCAGUUGCGCCGAAAGGCGCUCUGUAAUCAUUUAAAAGUAUUUUGAACGGACCUCUGUUUGACACGUCUGUU	75	41
		v2	GUUUCAGUUGCUGAAUUAUUUGGUAAACAGUACCAAAUAAUUA**AUG**CUCUGUAAUCAUUUAAAAGUAUUUUGAACGGACCUCUGUUUGACACGUCUGAAUAACUAAAAAUU	111	90
7	CdCas9	v1	ACUGGGGUUCAGGAAACUGAACCUCAGUAAGCAUUGGCUCGUUUCCA**AUG**UUGAUUGCUCCGCCGGUGCUCCUUAUUUUUAAGGGCGCCGGCUUUUAG	98	26
		v2	ACUGGGGUUCAGGAAACUGAACCUCAGUAAGCAUUGGCUCGUUUCCA**AUG**UUGAUUGCUCCGCCGGUGCUCCUUAUUUUUAAGGGCGCCGGCUUUAG	97	19
8	ClCas9	v1	GUUUUAGUCUCUGAAAAGAGACUAAAAUAAGUGGUUUUUGGUCAUCCACGCAGGGUUACAAUCCCUUUAAAACCAUUAAAAUUCAAAUAAACUAGGUUGUAUCAACUUAGUUU	113	36
9	PlCas9	v1	GCUGCGGAUUGCGGGAAAUCGCUUUUCGCAAGCAAAUUGACCCCUUGUGCGGGCUCGGCAUCCCAAGGUCAGCUGCCGGUUAUUAUCGAAAAGGCCCACCGCAAGCAGCGCGUGGGCCUUUU	122	13
		v2	GCUGCGGAUUGCGGGAAAUCGCUUUUCGCAAGCAAAUUGACCCCUUGUGCGGGCUCGGCAUCCCAAGGUCAGCUGCCGGUUAUUAUCGAAAAGGCCCACCGCAAGCAGCGCGUGGGCCUUUUUU	124	29
10	NcCas9	v1	GUUGUAGCUCCCAUUCUCGAAAGAGAACCGUUGCUACAAUAAGGCCGUCUGAAAAG**AUG**UGCCGCAACGCUCUGCCCCUUAAAGCUUCUGCUUUAAGGGGCAUCGUUU	108	88
		v2	GUUGUAGCUCCCAUUCUCGAAAGAGAACCGUUGCUACAAUAAGGCCGUCUGAAAAG**AUG**UGCCGCAACGCUCUGCCCCUUAAAGCUUCUGCUUUAAGGGGCAUCGUUUAUUUCGGUUAAAA**AUG**CCGUCUGAAACCGGUUUUUAGGUUUCAGACGGCAUUUU	162	83

11	SpaCas9	v1	GUUUUUGUACUCGAAAGAGCCUACAAAGAUAAGGCUUU**AUG**CCGAAUUCAA GCACCCC**AUG**UUUUGAC**AUG**AGGUGCUUUUAG	83	77
		v2	GUUUUUGUACUCGAAAGAGCCUACAAAGAUAAGGCUUU**AUG**CCGAAUUCAA GCACCCC**AUG**UUUUGAC**AUG**AGGUGCUUUAG	82	84
12	St3Cas9	v1	GUUUUAGAGCUGGGUACCCAGCGAGUUAAAAUAAGGCUUAGUCCGUACUCA ACUUGAAAAGGUGGCACCGAUUCGGUGUU	80	87
		v2	GUUUUAGAGCUGUGUUGUUUGUUAAAACAACACAGCGAGUUAAAAUAAGGC UUAGUCCGUACUCAACUUGAAAAGGUGGCACCGAUUCGGUGUU	94	87
	Name		Inserted RNA sequence	crRNA length (nt)[*2]	Repression efficiency (%)
1	AsCpf1 (AsCas12a)	v1	AAUUUCUACUCUUGUAGAUAGAUCAGGGCAAACAGAACUUUGA	43	90
2	FnCpf1 (FnCas12a)	v1	UAAUUUCUACUGUUGUAGAU	20	86
3	LbCpf1 (LbCas12a)	v1	UAAUUUCUACUAAGUGUAGAU	21	91
4	MbCpf1 (MbCas12a)	v1	AAAUUUCUACUGUUUGUAGAU	21	93
	Name		Inserted RNA sequence	sgRNA length (nt)[*2]	Repression efficiency (%)
1	AaCas12b	v1	GUCUAAAGGACAGAAUUUUUCAACGGGUGUGCCA**AUG**GCCACUUUCCAGGU GGCAAAGCCCGUUGAACUUCUCAAAAAGAACGCUCGCUCAGUGUUCUGACG UCGGAUCACUGAGCGAGCGAUCUGAGAAGUGGCAC	137	83
2	AkCas12b	v1	GUCGUCUAUAGGACGGCGAGGACAACGGGAAGUGCCA**AUG**UGCUCUUUCCA AGAGCAAACACCCCGUUGGCUUCAAG**AUG**ACCGCUCGAAAACGAGCGGUCU GAGAAGUGGCACUUC	117	91
		v2	UCGUCUAUAGGACGGCGAGGACAACGGGAAGUGCCA**AUG**UGCUCUUUCCAA GAGCAAACACCCCGUUGGCUUCAAG**AUG**ACCGCUCGCUCAGCGAUCUGACA ACGGAUCGCUGAGCGAGCGGUCUGAGAAGUGGCAC	137	78
3	BvCas12b	v1	GACCUAUAGGGUCA**AUG**AAUCUGUGCGUGUGCCAUAAGUAAUUAAAAAUUA CCCACCACAGGAUCAUCUUAUUUCAAAAGAAAUAAG**AUG**AUUGGCAC	98	85
	Name		Inserted RNA sequence	crRNA length (nt)[*2]	Repression efficiency (%)
1	PspCas13b	v1	GAGAUCAGGGCAAACAGAACUUUGACUCCCGUUGUGGAAGGUCCAGUUUUG AGGGGCUAUUACAAC	66	97
2	PguCas13b	v1	GAGAUCAGGGCAAACAGAACUUUGACUCCCGUUGGAUCUACCCUCUAUUUG AAGGGUACACACAAC	66	96
3	RanCas13b	v1	GAGAUCAGGGCAAACAGAACUUUGACUCCCGUUGGGACUGCUCUCACUUUG AAGGGUAUUCACAAC	66	83
4	CasRx (RfxCas13d)	v1	CAAGUAAACCCCUACCAACUGGUCGGGGUUUGAAACGAGAUCAGGGCAAAC AGAACUU	58	87
	Name		Inserted RNA sequence	sgRNA length (nt)[*2]	Repression efficiency (%)
1	PlmCasX	v1	GGCGCGUUUAUUCCAUUACUUUGGAGCCAGUCCCAGCGACU**AUG**UCGU**AUG** GACGAAGCGCUUAUUUAUCGGAGAGAAACCGAUAAGUAAAACGCAUCAAAG UCCUGCAGCAGAAAAUCAAA	122	63
		v2	GGCGCGUUUAUUCCAUUACUUUGGAGCCAGUCCCAGCGACU**AUG**UCGU**AUG** GACGAAGCGCUUAUUUAUCGGAGAGAAACCGAUAAGUAAAACGCAUCAAAG	102	66
2	Cas14a1	v1	CUUCACUGAUAAAGUGGAGAACCGCUUCACCAAAAGCUGUCCCUUAGGGGA UUAGAACUUGAGUGAAGGUGGGCUGCUUGCAUCAGCCUA**AUG**UCGAGAAGU GCUUUCUUCGGAAAGUAACCCUCGAAACAAAUUCAUUUGAAAGA**AUG**AAGG A**AUG**CAACCU	163	86
		v2	CUUCACUGAUAAAGUGGAGAACCGCUUCACCAAAAGCUGUCCCUUAGGGGA UUAGAACUUGAGUGAAGGUGGGCUGCUUGCAUCAGCCUA**AUG**UCGAGAAGU GCUUUCUUCGGAAAGUAACCCUCGAAACAAAUUCAUUUUUCCUCUCCAAUU CUGCACAAGAAAGUUGCAGAACCCGAAUAGACGA**AUG**AAGGA**AUG**CAACCU	204	78
	Name		Inserted RNA sequence	Aptamer length (nt)[*3]	Repression efficiency (%)[*3]
1	L7Ae	v1	GGGGCGUGAUCCGAAAGGUGACCC	24	98
2	MS2CP(V29I)	v1	GGGAGCAGGUGAGGAUCACCCAUCUGCCACGAGCGAGGUGAGGAUCACCCA UCUCGCUCGUGUUCCC	67	97
3	PP7CP	v1	GGGAGCUAAGGAGUUUAUAUGGAAACCCUUAGCCUGCUGCGUAAGGAGUUU AUAUGGAAACCCUUACGCAGCAGUUCCC	79	80

*1 For the PlmCasX-responsive switch (PlmCasX_gRNA 1), the spacer sequence described in the original report was used (Ref 20).
*2 Length of the sgRNA/crRNA used in this study.
*3 Data were referred from Ref 22.

Supplementary Table 3
Components of each 60 AND gate tested in Figure 6.

AND #	Input A	Input B	Cas protein C
1	PguCas13b	MbCas12a	PspCas13b
2	PguCas13b	SaCas9	PspCas13b
3	PguCas13b	AkCas12b	PspCas13b
4	PguCas13b	NcCas9	PspCas13b
5	MbCas12a	SaCas9	PspCas13b
6	MbCas12a	AkCas12b	PspCas13b
7	MbCas12a	NcCas9	PspCas13b
8	SaCas9	AkCas12b	PspCas13b
9	SaCas9	NcCas9	PspCas13b
10	AkCas12b	NcCas9	PguCas13b
11	PspCas13b	MbCas12a	PguCas13b
12	PspCas13b	SaCas9	PguCas13b
13	PspCas13b	AkCas12b	PguCas13b
14	PspCas13b	NcCas9	PguCas13b
15	MbCas12a	SaCas9	PguCas13b
16	MbCas12a	AkCas12b	PguCas13b
17	MbCas12a	NcCas9	PguCas13b
18	SaCas9	AkCas12b	PguCas13b
19	SaCas9	NcCas9	PguCas13b
20	AkCas12b	NcCas9	PguCas13b
21	PspCas13b	PguCas13b	MbCas12a
22	PspCas13b	SaCas9	MbCas12a
23	PspCas13b	AkCas12b	MbCas12a
24	PspCas13b	NcCas9	MbCas12a
25	PguCas13b	SaCas9	MbCas12a
26	PguCas13b	AkCas12b	MbCas12a
27	PguCas13b	NcCas9	MbCas12a
28	SaCas9	AkCas12b	MbCas12a
29	SaCas9	NcCas9	MbCas12a
30	AkCas12b	NcCas9	MbCas12a

AND #	Input A	Input B	Cas protein C
31	PspCas13b	PguCas13b	SaCas9
32	PspCas13b	MbCas12a	SaCas9
33	PspCas13b	AkCas12b	SaCas9
34	PspCas13b	NcCas9	SaCas9
35	PguCas13b	MbCas12a	SaCas9
36	PguCas13b	AkCas12b	SaCas9
37	PguCas13b	NcCas9	SaCas9
38	MbCas12a	AkCas12b	SaCas9
39	MbCas12a	NcCas9	SaCas9
40	AkCas12b	NcCas9	SaCas9
41	PspCas13b	PguCas13b	AkCas12b
42	PspCas13b	MbCas12a	AkCas12b
43	PspCas13b	SaCas9	AkCas12b
44	PspCas13b	NcCas9	AkCas12b
45	PguCas13b	MbCas12a	AkCas12b
46	PguCas13b	SaCas9	AkCas12b
47	PguCas13b	NcCas9	AkCas12b
48	MbCas12a	SaCas9	AkCas12b
49	MbCas12a	NcCas9	AkCas12b
50	SaCas9	NcCas9	AkCas12b
51	PspCas13b	PguCas13b	NcCas9
52	PspCas13b	MbCas12a	NcCas9
53	PspCas13b	SaCas9	NcCas9
54	PspCas13b	AkCas12b	NcCas9
55	PguCas13b	MbCas12a	NcCas9
56	PguCas13b	SaCas9	NcCas9
57	PguCas13b	AkCas12b	NcCas9
58	MbCas12a	SaCas9	NcCas9
59	MbCas12a	AkCas12b	NcCas9
60	SaCas9	AkCas12b	NcCas9

Supplementary Table 4

Statistical analysis related to Figure 6F.

Tukey's multiple comparison tests were performed based on the experiments in Fig. 6F (Apoptosis regulation with AND gate circuits). The values in bold indicate statistical significance (*P* < 0.05).

Tukey's multiple comparison test	*P*-values in apoptosis regulatory AND gates			
Sate-State	Pgu_Sa_Psp Apoptotic	Pgu_Sa_Psp Dead	Psp_Sa_Ak Apoptotic	Psp_Sa_Ak Dead
[0,1]-[0,0]	0.9712	0.9866	0.9402	0.9942
[1,0]-[0,0]	0.5911	0.7852	0.7346	0.9113
[1,1]-[0,0]	**0.00004**	**0.008**	**0.0008**	**0.0051**
[1,0]-[0,1]	0.824	0.9264	0.9631	0.976
[1,1]-[0,1]	**0.0001**	**0.0122**	**0.0015**	**0.007**
[1,1]-[1,0]	**0.0001**	**0.0278**	**0.0026**	**0.0118**

Supplementary Table 5
Key transfected plasmids used in this study.

Description	Original name	Benchling link	Short name	Figure
OFF switch	pGluc-Sp_gRNA-EGFP	https://benchling.com/s/seq-YB5Dr4uWTfanMOKvIUsY	SpCas9_gRNA/ Sp_gRNA	Fig 1B, 1C, 1E, 3B, 3D, 4, Fig S2, S3A, S3B, S7A, S12, S13
OFF switch	pGluc-Sa_gRNA-EGFP	https://benchling.com/s/seq-M1VrldwdMbyqmlAdm2TA	SaCas9_gRNA/ Sa_gRNA	Fig 1E, 3F, 4, Fig S2, S13
OFF switch	pGluc-Cj_gRNA-EGFP	https://benchling.com/s/seq-i683ARLbCYEjQdq5Cnh0	CjCas9_gRNA	Fig 1E, 4, Fig S2, S6C, S7B, S13
OFF switch	pGluc-St1_gRNA-EGFP	https://benchling.com/s/seq-TskVXFHofl9E2pxSQWkM	St1Cas9_gRNA	Fig 1E, 4, Fig S2, S13
OFF switch	pFn_gRNA_v1-EGFP	https://benchling.com/s/seq-ovf2dJM05eLri2RCfAHh	FnCas9_gRNA1	Fig 1E, Fig S2
OFF switch	pFn_gRNA_v2-EGFP	https://benchling.com/s/seq-m3xNCSEq42TLjiLL293S	FnCas9_gRNA2	Fig 1E, 4, Fig S2, S13
OFF switch	pCd_gRNA_v1-EGFP	https://benchling.com/s/seq-K6uhc21sAPxxOEK6PZuw	CdCas9_gRNA1	Fig 1E, 4, Fig S2, S13
OFF switch	pCd_gRNA_v2-EGFP	https://benchling.com/s/seq-KZVPW44Dk4V6u4zafopV	CdCas9_gRNA2	Fig 1E, Fig S2
OFF switch	pCl_gRNA-EGFP	https://benchling.com/s/seq-H4UxWLOhgNvA0YR4Szip	ClCas9_gRNA	Fig 1E, 4, Fig S2, S13
OFF switch	pPl_gRNA_v1-EGFP	https://benchling.com/s/seq-VfDteXn2JGE7dYPfy9D3	PlCas9_gRNA1	Fig 1E, Fig S2
OFF switch	pPl_gRNA_v2-EGFP	https://benchling.com/s/seq-wilfLbHEW4cECDThdcwt	PlCas9_gRNA2	Fig 1E, 4, Fig S2, S13
OFF switch	pNc_gRNA_v1-EGFP	https://benchling.com/s/seq-9GsfCiNp0dlL6B6UFmpP	NcCas9_gRNA1	Fig 1E, 4, Fig S2, S13
OFF switch	pNc_gRNA_v2-EGFP	https://benchling.com/s/seq-oF6McTKt84TdwsJ5Wt1H	NcCas9_gRNA2	Fig 1E, Fig S2
OFF switch	pSpa_gRNA_v1-EGFP	https://benchling.com/s/seq-PRk8eHaoV3JHCtXrX3BE	SpaCas9_gRNA1	Fig 1E, Fig S2
OFF switch	pSpa_gRNA_v2-EGFP	https://benchling.com/s/seq-Oh0lQ13b1xRVbmpRBqYH	SpaCas9_gRNA2	Fig 1E, 4, Fig S2, S13
OFF switch	pGluc-St3_gRNA_v1-EGFP	https://benchling.com/s/seq-N17o8zbHO751wm0LPAsa	St3Cas9_gRNA1	Fig 1E, Fig S2
OFF switch	pGluc-St3_gRNA_v2-EGFP	https://benchling.com/s/seq-BwCIYQ2YCuw2O3GdfvUV	St3Cas9_gRNA2	Fig 1E, 4, Fig S2, S13
OFF switch	pGluc-AsCas12a_crRNA-EGFP	https://benchling.com/s/seq-glNfU5jZtiKihoYUIYbg	AsCas12a_crRNA	Fig 1E, 4, Fig S2, S10, S13
OFF switch	pFnCas12a_crRNA-EGFP	https://benchling.com/s/seq-V8aaKQn6IXZBvwOrjF4e	FnCas12a_crRNA	Fig 1E, 4, Fig S2, S13
OFF switch	pLbCas12a_crRNA-EGFP	https://benchling.com/s/seq-AjWByTr164WVX6iEujJH	LbCas12a_crRNA	Fig 1E, 4, Fig S2, S13
OFF switch	pMbCas12a_crRNA-EGFP	https://benchling.com/s/seq-hbYhHvSP2IG6F4WpK4IN	MbCas12a_crRNA	Fig 1E, 4, Fig S2, S13
OFF switch	pAaCas12b_sgRNA-EGFP	https://benchling.com/s/seq-0ah7ki0EgntdfZahBDPU	AaCas12a_crRNA	Fig 1E, 4, Fig S2, S13
OFF switch	pAkCas12b_sgRNA_v1-EGFP	https://benchling.com/s/seq-7ZiHjayLdyOwjiBwW9qe	AkCas12b_gRNA1	Fig 1E, 4, Fig S2, S13
OFF switch	pAkCas12b_sgRNA_v2-EGFP	https://benchling.com/s/seq-WhxCmn2hJG2pYh5T4JaK	AkCas12b_gRNA2	Fig 1E, Fig S2
OFF switch	pBvCas12b_sgRNA-EGFP	https://benchling.com/s/seq-ERy3edQQ4oRn27cBoE1F	BvCas12b_gRNA	Fig 1E, 4, Fig S2, S13
OFF switch	pGluc-Psp_crRNA-EGFP	https://benchling.com/s/seq-WJlEdGEGMXhOOPGzuGHZ	PspCas13b_crRNA / Layer0 (circuit)	Fig 1E, 4, 7B, Fig S2, S6A, S13
OFF switch	pGluc-Pgu_crRNA-EGFP	https://benchling.com/s/seq-q7r7cKeYiylzuEg4k7bP	PguCas13b_crRNA	Fig 1E, 4, Fig S2, S6B, S13
OFF switch	pGluc-Ran_crRNA-EGFP	https://benchling.com/s/seq-dR5ZevWPdwURmwnkXMsA	RanCas13b_crRNA	Fig 1E, 4, Fig S2, S13
OFF switch	pGluc-CasRX_crRNA-EGFP	https://benchling.com/s/seq-E6RZl3LyVrpYHNZuhz06	CasRx_crRNA	Fig 1E, 4, Fig S2, S13
OFF switch	pPlmCasX_gRNA-EGFP	https://benchling.com/s/seq-CN4z7nmpzAYbyQPomM4H	PlmCasX_gRNA1	Fig 1E, Fig S2

OFF switch	pPlmCasX_gRNA(dSpacer)-EGFP	https://benchling.com/s/seq-XSQeSp220dxaYoc5BkL9	PlmCasX_gRNA2	Fig 1E, 4, Fig S2, S13
OFF switch	pCas14a1_sgRNA1-EGFP	https://benchling.com/s/seq-nL3klfKUsOsPT2W8iuz7	Cas14a1_gRNA1	Fig 1E, 4, Fig S2, S13
OFF switch	pCas14a1_sgRNA2-EGFP	https://benchling.com/s/seq-oIcaD1LAx1CS6wGriPSc	Cas14a1_gRNA2	Fig 1E, Fig S2
OFF switch	pGluc-Nm_gRNA-EGFP	https://benchling.com/s/seq-9ShtAQING48KU8WRPZI0	NmCas9_gRNA_v0	Fig 1E, Fig S2
OFF switch	pGluc-Nm_gRNA-STOP-EGFP	https://benchling.com/s/seq-I3yoBcMcdBdqRQRUwmXg	NmCas9_gRNA_v1	Fig 1E, Fig S2
OFF switch	pGluc-Nm_gRNA_v2-EGFP	https://benchling.com/s/seq-4QVuutAH6V2urfJlIMRy	NmCas9_gRNA_v2	Fig 1E, Fig S2
OFF switch	pGluc-Nm_gRNA_v3-EGFP	https://benchling.com/s/seq-wbDRU1SvlenI79ZS1H2F	NmCas9_gRNA_v3	Fig 1E, Fig S2
OFF switch	pNm_gRNA_v4-EGFP	https://benchling.com/s/seq-b1iBbNY6afS8lW4wlZMq	NmCas9_gRNA_v4	Fig 1E, Fig S2
OFF switch	pNm_gRNA_v5-EGFP	https://benchling.com/s/seq-mPrZo0zK3tP8fOo0fXtN	NmCas9_gRNA_v5	Fig 1E, Fig S2
OFF switch	pNm_gRNA_v6-EGFP	https://benchling.com/s/seq-tHODiWmkQAncUaHAP2Et	NmCas9_gRNA_v6	Fig 1E, Fig S2
OFF switch	pNm_gRNA_v7-EGFP	https://benchling.com/s/seq-CXq80mUzT3gHGyDvJXiX	NmCas9_gRNA_v7	Fig 1E, 4, Fig S2, S13
**Description**	**Original name**	**Benchling link and Addgene #**	**Short name**	**Figure**
Trigger	pcDNA3.1-SpCas9	https://benchling.com/s/seq-EmEEkWHXs3Jf4rcdpaTg	SpCas9/WT	Fig 1B, 1C, 1E, 2, 3B, 3D, 4, Fig S2, S3A, S3B, S7A, S8, S12, S13, S14
Trigger	pcDNA3.1+-SaCas9	https://benchling.com/s/seq-Sj4R81pj6RXIZ6Jly9p4	SaCas9	Fig 1E, 2, 3F, 4, 6B, 6F, 7B, 7D, Fig S2, S8, S13, S14, S17, S18
Trigger	pcDNA3.1+-CjCas9	https://benchling.com/s/seq-6eXuV9JcFQeQbmYwfJX8	CjCas9	Fig 1E, 2, 4, 7D, Fig S2, S6C, S7B, S8, S13, S14
Trigger	pcDNA3.1+-NmCas9	https://benchling.com/s/seq-Wfg75vjX32YQQJkiyuYE	NmCas9	Fig 1E, 2, 4, Fig S2, S8, S13, S14
Trigger	pcDNA3.1+-st1Cas9	https://benchling.com/s/seq-VRWF7oQowjQbAvG8mYce	St1Cas9	Fig 1E, 2, 4, Fig S2, S8, S13, S14
Trigger	pcDNA3.1+-FnCas9	https://benchling.com/s/seq-T1yYZahdAytxkLbQf5Jw	FnCas9	Fig 1E, 2, 4, Fig S2, S8, S13, S14
Trigger	pcDNA3.1+-CdCas9	https://benchling.com/s/seq-kC3slahy2rNyCbP39Uls	CdCas9	Fig 1E, 2, 4, Fig S2, S8, S13, S14
Trigger	pcDNA3.1+-ClCas9	https://benchling.com/s/seq-QMBrgPUM4wQ6nJFQp2Jv	ClCas9	Fig 1E, 2, 4, Fig S2, S8, S13, S14
Trigger	pcDNA3.1+-PlCas9	https://benchling.com/s/seq-ibuHG9biYiK2zC93XPUq	PlCas9	Fig 1E, 2, 4, Fig S2, S8, S13, S14
Trigger	pcDNA3.1+-NcCas9	https://benchling.com/s/seq-ifoU1HSjWp7QaJ2rnz4O	NcCas9	Fig 1E, 2, 4, 6B, Fig S2, S8, S13, S14
Trigger	pcDNA3.1+-SpaCas9	https://benchling.com/s/seq-hmk13W570AKm7GQ0roes	SpaCas9	Fig 1E, 2, 4, Fig S2, S8, S13, S14
Trigger	pcDNA3.1+-St3Cas9	https://benchling.com/s/seq-2q6Vcj3AwAHrO3ZXoP3Y	St3Cas9	Fig 1E, 2, 4, Fig S2, S8, S13, S14
Trigger	pcDNA3.1+-AsCpf1	https://benchling.com/s/seq-7cpBK5lcPWKG0wk88fqZ	AsCas12a	Fig 1E, 2, 4, Fig S2, S8, S10, S13, S14
Trigger	pcDNA3.1-hMbCpf1	Addgene ID #69986	MbCas12a / Layer 4	Fig 1E, 2, 4, 6B, 7B Fig S2, S8, S13, S14
Trigger	pcDNA3.1+-AaCas12b	https://benchling.com/s/seq-Cg0Aurq89nwhVyoOf7cz	AaCas12b	Fig 1E, 2, 4, Fig S2, S8, S13, S14
Trigger	pcDNA3.1-hFnCpf1	Addgene ID #69976	FnCas12a	Fig 1E, 2, 4, Fig S2, S8, S13, S14
Trigger	pcDNA3.1-hLbCpf1	Addgene ID #69988	LbCas12a	Fig 1E, 2, 4, Fig S2, S8, S13, S14
Trigger	pcDNA3.1+-AkCas12b	https://benchling.com/s/seq-9CJWprWiG5TviPN6Zh4R	AkCas12b	Fig 1E, 2, 4, 6B, Fig S2, S8, S13, S14
Trigger	pcDNA3.1+-BvCas12b	https://benchling.com/s/seq-lwRpPTKuL6fB3m0XNzK6	BvCas12b	Fig 1E, 2, 4, Fig S2, S8, S13, S14

Trigger	pcDNA3.1-PspCas13b(WT)-NES-myc-His6	https://benchling.com/s/seq-X0bNQN8K5SsJBN6F3OOX	PspCas13b	Fig 1E, 2, 4, 6B, 6F, 7B, 7F, Fig S2, S6A, S8, S13, S14, S18
Trigger	pcDNA3.1+-PguCas13b-NES	https://benchling.com/s/seq-BPSiSwCOpWBOxS0GGgcD	PguCas13b	Fig 1E, 2, 4, 6B, 6F, 7B, 7F, Fig S2, S6B, S8, S13, S14, S18
Trigger	pcDNA3.1+-RanCas13b-NES	https://benchling.com/s/seq-j8EjvpKgsQntC2aumCLE	RanCas13b	Fig 1E, 2, 4, Fig S2, S8, S13, S14
Trigger	pcDNA3.1+-CasRx	https://benchling.com/s/seq-9sXxgMXbXXXfw9qoAtLo	CasRx	Fig 1E, 2, 4, Fig S2, S8, S13, S14
Trigger	pcDNA3.1+-PlmCasX	https://benchling.com/s/seq-iwnSWt8Fk44t9U9QBnqa	PlmCasX	Fig 1E, 2, 4, Fig S2, S8, S13, S14
Trigger	pcDNA3.1+-Cas14a1	https://benchling.com/s/seq-omwxft6FEDKBWlKxejS4	Cas14a1	Fig 1E, 2, 4, Fig S2, S8, S13, S14
**Description**	**Original name**	**Benchling link**	**Short name**	**Figure**
Trigger	pcDNA3.1-SpCas9(D10A)	https://benchling.com/s/seq-rglwwC4Z2fEgTNhxNCmX	D10A	Fig S3A
Trigger	pcDNA3.1-SpCas9(D10A_H840)	https://benchling.com/s/seq-oTpkJKDiTUBXWxa45FDX	D10A, H840A	Fig S3A
Trigger	pcDNA3.1-SpCas9(dNLS)	https://benchling.com/s/seq-xIMuZsE3gLNDmcQDstVq	ΔNLS	Fig S3A
Acr	pcDNA3.1+-AcrIIA4	https://benchling.com/s/seq-gGe8r2DZoDQvxqDhar3j	AcrIIA4	Fig S3B
**Description**	**Original name**	**Benchling link**	**Short name**	**Figure**
Trigger	pcDNA3.1+-AsCpf1(H800A)	https://benchling.com/s/seq-9U2OSQeiLx0biaTx8INe	AsCas12a(H800A)	Fig S9
**Description**	**Original name**	**Benchling link**	**Short name**	**Figure**
ON switch	pNMD-ON-Gluc-Sp_gRNA-EGFP	https://benchling.com/s/seq-rBiY9xY8lxCQlmCDzb2v	SpCas9_gRNA	Fig 2, Fig S8, S14
ON switch	pNMD-ON-Gluc-Sa_gRNA	https://benchling.com/s/seq-Rkh4phoiHfg2TfGdl4LP	SaCas9_gRNA	Fig 2, 7D, Fig S8, S14, S17
ON switch	pNMD-ON-Gluc-Cj_gRNA	https://benchling.com/s/seq-ytclQFC5hKdeTzpgVAgg	CjCas9_gRNA	Fig 2, 7D Fig S8, S14
ON switch	pNMD-ON-Gluc-St1_gRNA	https://benchling.com/s/seq-edpM2PVwBHJPhFaMwMGq	St1Cas9_gRNA	Fig 2, Fig S8, S14
ON switch	pNMD-ON-Gluc_Nm_gRNA_v1	https://benchling.com/s/seq-j8yTztzbcE5l6BnnaYcV	NmCas9_gRNA1	Fig 2, Fig S8
ON switch	pNMD-ON-Gluc_Nm_gRNA_v2	https://benchling.com/s/seq-4JkdlEgacfZVliRF9dFW	NmCas9_gRNA2	Fig 2, Fig S8
ON switch	pNMD-ON-Gluc_Nm_gRNA_v3	https://benchling.com/s/seq-AYCFLt1uYmg1tnfU7TLk	NmCas9_gRNA3	Fig 2, Fig S8, S14
ON switch	pNMD-ON-Gluc_Nm_gRNA_v4	https://benchling.com/s/seq-lg2jkJ3G8H1fd4MMQ4CO	NmCas9_gRNA4	Fig 2, Fig S8
ON switch	pNMD-ON-Gluc_Nm_gRNA_v5	https://benchling.com/s/seq-fXdYnMeOXbnz6Whw4Jed	NmCas9_gRNA5	Fig 2, Fig S8
ON switch	pNMD-ON-Gluc_Nm_gRNA_v6	https://benchling.com/s/seq-VX81PClbzWVwRpJdbQNJ	NmCas9_gRNA6	Fig 2, Fig S8
ON switch	pNMD-ON-Gluc_Nm_gRNA_v7	https://benchling.com/s/seq-gzSPC4IFm9ll9ShoyHlr	NmCas9_gRNA7	Fig 2, Fig S8
ON switch	pNMD-ON-Fn_gRNA_v1	https://benchling.com/s/seq-UpTK1Av0CTE5Qzgf5OSR	FnCas9_gRNA1	Fig 2, Fig S8
ON switch	pNMD-ON-Fn_gRNA_v2	https://benchling.com/s/seq-wnexJ24lCiNDjWylaPjl	FnCas9_gRNA2	Fig 2, Fig S8, S14
ON switch	pNMD-ON-Cd_gRNA_v1	https://benchling.com/s/seq-aZ03Yf3zGnKi9y1qrP35	CdCas9_gRNA1	Fig 2, Fig S8
ON switch	pNMD-ON-Cd_gRNA_v2	https://benchling.com/s/seq-Mssi3187k0LiwSpbSY1g	CdCas9_gRNA2	Fig 2, Fig S8, S14
ON switch	pNMD-ON-Cl_gRNA	https://benchling.com/s/seq-gkRuH0CD6l8VFkLfGTuN	ClCas9_gRNA	Fig 2, Fig S8, S14
ON switch	pNMD-ON-Pl_gRNA_v1	https://benchling.com/s/seq-neQ5XOaKZayZxaEnBpBJ	PlCas9_gRNA1	Fig 2, Fig S8
ON switch	pNMD-ON-Pl_gRNA_v2	https://benchling.com/s/seq-uo12MN8p9ELYe4KBwKPy	PlCas9_gRNA2	Fig 2, Fig S8, S14
ON switch	pNMD-ON-Nc_gRNA_v1	https://benchling.com/s/seq-57djv04vQeZYOqLdpZB9	NcCas9_gRNA1	Fig 2, Fig S8, S14

ON switch	pNMD-ON-Nc_gRNA_v2	https://benchling.com/s/seq-5Kd4BkOffc6EGynJb7Xf	NcCas9_gRNA2	Fig 2, Fig S8
ON switch	pNMD-ON-Spa_gRNA_v1	https://benchling.com/s/seq-fnoQ3X1Qs6sCtgmAhZQ6	SpaCas9_gRNA1	Fig 2, Fig S8
ON switch	pNMD-ON-Spa_gRNA_v2	https://benchling.com/s/seq-OmmTbhPsOewdpW8J7vJh	SpaCas9_gRNA2	Fig 2, Fig S8, S14
ON switch	pNMD-ON-St3_gRNA_v1	https://benchling.com/s/seq-vvJqP4bzkFr0jzUDum9m	St3Cas9_gRNA1	Fig 2, Fig S8, S14
ON switch	pNMD-ON-St3_gRNA_v2	https://benchling.com/s/seq-0hRIB3bEUrvRH6pStcVP	St3Cas9_gRNA2	Fig 2, Fig S8
ON switch	pNMD-ON-Gluc-AsCas12a_crRNA	https://benchling.com/s/seq-uMWXFaSnWI4Tj569dVR3	AsCas12a_crRNA	Fig 2, Fig S8, S14
ON switch	pNMD-ON-FnCas12a_crRNA	https://benchling.com/s/seq-zCLlzSzy9FGZVE8LqSGw	FnCas12a_crRNA	Fig 2, Fig S8, S14
ON switch	pNMD-ON-LbCas12a_crRNA	https://benchling.com/s/seq-097cK15zRAFJnNdSzJrU	LbCas12a_crRNA	Fig 2, Fig S8, S14
ON switch	pNMD-ON-MbCas12a_crRNA	https://benchling.com/s/seq-2MgLIE9wFI3d5ljtMnbC	MbCas12a_crRNA	Fig 2, Fig S8, S14
ON switch	pNMD-ON-AaCas12b_sgRNA	https://benchling.com/s/seq-omJehIz6xnx7yEdD9GMw	AaCas12a_crRNA	Fig 2, Fig S8, S14
ON switch	pNMD-ON-AkCas12b_sgRNA_v1	https://benchling.com/s/seq-eNnHdLLBxiWLCq3zFjEl	AkCas12b_gRNA1	Fig 2, Fig S8, S14
ON switch	pNMD-ON-AkCas12b_sgRNA_v2	https://benchling.com/s/seq-9AOIST8qZ7TUMkhdqs9K	AkCas12b_gRNA2	Fig 2, Fig S8
ON switch	pNMD-ON-BvCas12b_sgRNA	https://benchling.com/s/seq-y5azslDwpaymVWAunhno	BvCas12b_gRNA	Fig 2, Fig S8, S14
ON switch	pNMD-ON-Gluc-Psp_crRNA-EGFP	https://benchling.com/s/seq-6A2RLRkhHstAACjndh3T	PspCas13b_crRNA	Fig 2, Fig S8, S14
ON switch	pNMD-ON-Gluc-Pgu_crRNA	https://benchling.com/s/seq-A8zYUC0tnz18biHZwwih	PguCas13b_crRNA	Fig 2, Fig S8, S14
ON switch	pNMD-ON-Gluc_Ran_crRNA	https://benchling.com/s/seq-INRGbAowneL4z9J9NlpK	RanCas13b_crRNA	Fig 2, Fig S8, S14
ON switch	pNMD-ON-Gluc-CasRx_crRNA	https://benchling.com/s/seq-bHrydKbzUXtMT2df0vvF	CasRx_crRNA	Fig 2, Fig S8, S14
ON switch	pNMD-ON-PlmCasX_gRNA	https://benchling.com/s/seq-1ASZJgt3yMozw41vGMC4	PlmCasX_gRNA1	Fig 2, Fig S8, S14
ON switch	pNMD-ON-PlmCasX_gRNA(dSpacer)	https://benchling.com/s/seq-2vhdu4FdsBUepnpg8sbH	PlmCasX_gRNA2	Fig 2, Fig S8
ON switch	pNMD-ON-Cas14a1_sgRNA_v1	https://benchling.com/s/seq-X7M8REytCChUfrThKnd0	Cas14a1_gRNA1	Fig 2, Fig S8, S14
ON switch	pNMD-ON-Cas14a1_sgRNA_v2	https://benchling.com/s/seq-gcwfVBOq93RuFqMvzOZm	Cas14a1_gRNA2	Fig 2, Fig S8
**Description**	**Original name**	**Benchling link**	**Short name**	**Figure**
Trigger	pcDNA3.1-SpCas9(1-713)	https://benchling.com/s/seq-WhnSX12uuWktk1Ptnl6N	SpCas9(1-713)	Fig S12
Trigger	pcDNA3.1-SpCas9(714-1368)	https://benchling.com/s/seq-EgutSzE4sOjkDFpvAga6	SpCas9(714-1368)	Fig S12
Trigger	pcDNA3.1-SpCas9(1-713)-N_intein	https://benchling.com/s/seq-LznvtjWCo3gRuQTQLrFV	N-Cas9	Fig 3B
Trigger	pcDNA3.1-C_intein-SpCas9(714-1368)	https://benchling.com/s/seq-IQZPP14tzFCrLOAcMehv	C-Cas9	Fig 3B
Trigger	pcDNA3.1-SpCas9(1-713)-(GGGGS)3-DmrA	https://benchling.com/s/seq-dv1jMt9Ay11JeCJLUQVH		Fig 3D
Trigger	pcDNA3.1-DmrC-(GGGGS)3-SpCas9(714-1368)	https://benchling.com/s/seq-Fm4hweFErSPt3TEBtD2O		Fig 3D
**Description**	**Original name**	**Benchling link**	**Short name**	**Figure**
Acr	pcDNA3.1+-AcrIIC2_Nm	https://benchling.com/s/seq-q7VMJRLxbI80KzAUR0GI	AcrIIC2	Fig 3F
**Description**	**Original name**	**Benchling link**	**Short name**	**Figure**
OFF switch	pGluc-Sa_gRNA-tagRFP	https://benchling.com/s/seq-CdGyAKcK4BLcaxnwoJqB		Fig S17

Description	Original name	Benchling link	Short name	Figure
TX-TL	pGluc-Sp_gRNA-tagRFP	https://benchling.com/s/seq-lDi3fB9tIGStqMMVnkq5	pSp_gRNA-RFP	Fig 5
TX-TL	pTRE-Tight-hmAG1	https://benchling.com/s/seq-2cqCEKIUhTzatrtUhv0P	pTRE-hmAG1	Fig 5
TX-TL	SP-dCas9-VPR	https://benchling.com/s/seq-ZEIwRvTTCW7jt0rU9sK6	dSpCas9-VPR	Fig 5, 8
TX-TL	pHL-gRNA[TRE new]-iRFP-RIH	https://benchling.com/s/seq-q7JGwy2sJVyYMauIUoN3	gRNA	Fig 5
**Description**	**Original name**	**Benchling link**	**Short name**	**Figure**
60 AND	pAkCas12b_sgRNA_v1-hMbCpf1	https://benchling.com/s/seq-nCZ2RO20R6AumIh4YjgK		Fig 6B
60 AND	pAkCas12b_sgRNA_v1-NcCas9	https://benchling.com/s/seq-1a7DesFIHNILsHpSu4w9		Fig 6B
60 AND	pAkCas12b_sgRNA_v1-PguCas13b-NES	https://benchling.com/s/seq-B67ojjSjELQKJys2zkGN		Fig 6B
60 AND	pAkCas12b_sgRNA_v1-PspCas13b-NES	https://benchling.com/s/seq-qz4qIieBfHwUzOWjC5e0		Fig 6B
60 AND	pAkCas12b_sgRNA_v1-SaCas9	https://benchling.com/s/seq-bzPeWMvq81ItvXucSkKQ		Fig 6B
60 AND	pGluc-Pgu_crRNA-AkCas12b	https://benchling.com/s/seq-9jNJRU5L8trZNL9Ea1Fk		Fig 6B
60 AND	pGluc-Pgu_crRNA-hMbCpf1	https://benchling.com/s/seq-K4EDhN81LGr1ZKzR7Zv4		Fig 6B
60 AND	pGluc-Pgu_crRNA-NcCas9	https://benchling.com/s/seq-aLCsxbyWI32xJbcDvUvE		Fig 6B
60 AND	pGluc-Pgu_crRNA-PspCas13b	https://benchling.com/s/seq-SjvgeWN3ybsNDiRJL3bl		Fig 6B, 6F, Fig S18
60 AND	pGluc-Pgu_crRNA-SaCas9	https://benchling.com/s/seq-xwARaxvsdDQ0dPAR5yLI		Fig 6B
60 AND	pGluc-Psp_crRNA-AkCas12b	https://benchling.com/s/seq-6uVhkXlqnt3fl0fTPK9b		Fig 6B, 6F, Fig S18
60 AND	pGluc-Psp_crRNA-hMbCpf1	https://benchling.com/s/seq-exsrOWx6k6to67kEyp9l		Fig 6B
60 AND	pGluc-Psp_crRNA-NcCas9	https://benchling.com/s/seq-RfV6xht046zxIi5hD6kJ		Fig 6B
60 AND	pGluc-Psp_crRNA-PguCas13b-NES	https://benchling.com/s/seq-ErjJA7ofbgNchGonVhRw		Fig 6B
60 AND	pGluc-Psp_crRNA-SaCas9	https://benchling.com/s/seq-vMQG9zSxZXBfRrJbHaUu		Fig 6B
60 AND	pGluc-Sa_gRNA-AkCas12b	https://benchling.com/s/seq-gsFRzqnDG9GMjHBUxcXK		Fig 6B, 6F, Fig S18
60 AND	pGluc-Sa_gRNA-MbCas12a	https://benchling.com/s/seq-pcMlOudTctmDddOgJqYx		Fig 6B
60 AND	pGluc-Sa_gRNA-NcCas9	https://benchling.com/s/seq-PKfTqfWkEK0qDtiR6NaT		Fig 6B
60 AND	pGluc-Sa_gRNA-PguCas13b-NES	https://benchling.com/s/seq-1nJKeWZjZPKnwLvLAXw7		Fig 6B
60 AND	pGluc-Sa_gRNA-PspCas13b-NES	https://benchling.com/s/seq-vZdLDuRUj7lEkoSsrUus		Fig 6B, 6F, Fig S18
60 AND	pMbCas12a_crRNA-AkCas12b	https://benchling.com/s/seq-ij4dn2iIGnGvaZlxhdfJ		Fig 6B
60 AND	pMbCas12a_crRNA-NcCas9	https://benchling.com/s/seq-blf5FyEY59ZpzSoStpet		Fig 6B
60 AND	pMbCas12a_crRNA-PguCas13b-NES	https://benchling.com/s/seq-3xdNZDrleFaTH8dagqKG		Fig 6B
60 AND	pMbCas12a_crRNA-PspCas13b-NES	https://benchling.com/s/seq-qHKPavIjaz79DIq4Mk5f		Fig 6B
60 AND	pMbCas12a_crRNA-SaCas9	https://benchling.com/s/seq-n012CwEHH9uvfaQ0gvj5		Fig 6B
60 AND	pNc_gRNA_v1-AkCas12b	https://benchling.com/s/seq-uM9EVPlutz8XRqTR19bt		Fig 6B
60 AND	pNc_gRNA_v1-MbCpf1	https://benchling.com/s/seq-PY2fTqoxv4KsMFs6I2GF		Fig 6B

60 AND	pNc_gRNA_v1-PguCas13b-NES	https://benchling.com/s/seq-HdyvKPFRGxGzxA18WgRv		Fig 6B
60 AND	pNc_gRNA_v1-PspCas13b-NES	https://benchling.com/s/seq-QHZAHBtl1644ZiJJs246		Fig 6B
60 AND	pNc_gRNA_v1-SaCas9	https://benchling.com/s/seq-uO9Q7cbMhicDBiKJg0l8		Fig 6B
**Description**	**Original name**	**Benchling link**	**Short name**	**Figure**
Half-subtractor	pSa_IgRNA_a-CMVmin-Gluc_Sp_gRNA-tagBFP	https://benchling.com/s/seq-VNL3h459BChedQi2fG2A?m=slm-ONQM9WZqNR6RsVBWvzHJ		Fig 8
Half-subtractor	pSp_IgRNA_ax2-CMVmin-Gluc_Sa_gRNA-tagBFP-Triplex-HHR-Sp_gRNA[TRE]-HDVR	https://benchling.com/s/seq-KJyZz9cqwZRP7eApIY1X?m=slm-XQdF0YVyzRXOXywEqlIY		Fig 8
Half-subtractor	pSp_IgRNA_ax2-CMVmin-Gluc_Sa_gRNA-tagBFP	https://benchling.com/s/seq-KpJNkbiOwhNvphA7kTqG		Fig 8
Half-subtractor	pTRE-Tight-Gluc_Sp_gRNA-hmAG1	https://benchling.com/s/seq-RtHU0UdngTZHkwAhHqPP		Fig 8
Half-subtractor	pcDNA3.1+-dSaCas9-VPR	https://benchling.com/s/seq-VOVsrV43YHMX6DoRTtEC		Fig 8
Half-subtractor	pHL-Sa_IgRNA_a-iRFP-RIH	https://benchling.com/s/seq-Ay5dnk1zINPfqphQkY9X		Fig 8
Half-subtractor	pHL-Sp_IgRNA_a-iRFP-RIH	https://benchling.com/s/seq-BNfF7C046rnblBDNHpsC		Fig 8
**Description**	**Original name**	**Benchling link**	**Short name**	**Figure**
Apoptosis	pGluc-Psp_crRNA-hBax	https://benchling.com/s/seq-Z9dVEV6E89zkP5n9usnZ?m=slm-C7SxYot6Elupe56WmrlC		Fig 6F, Fig S18
Apoptosis	pAkCas12b_sgRNA_v1-hBax	https://benchling.com/s/seq-cdC2ICkaYtvtQRUv68ta?m=slm-ZKVPSfhwcEQFtKXaSWJ3		Fig 6F, Fig S18
**Description**	**Original name**	**Benchling link**	**Short name**	**Figure**
NOR gate	p(Gluc-Psp_crRNA)-(Gluc-Pgu_crRNA)-EGFP	https://benchling.com/s/seq-IADPDDHSzgq0Qjfog4ZM?m=slm-g7RJBS6BF2o3Q05smLPn	Psp_Pgu	Fig 7F
NOR gate	p(Gluc-Pgu_crRNA)-(Gluc-Psp_crRNA)-EGFP	https://benchling.com/s/seq-XuNmnrBBDFeSYuKetUPU?m=slm-5ajOQKyPOX4BYZ86xU7G	Pgu_Psp	Fig 7F
**Description**	**Original name**	**Benchling link**	**Short name**	**Figure**
Cascade OFF	pMbCas12a_crRNA-SaCas9	https://benchling.com/s/seq-n012CwEHH9uvfaQ0gvj5	Layer3	Fig 7B
Cascade OFF	pGluc-Sa_gRNA-PguCas13b-NES	https://benchling.com/s/seq-1nJKeWZjZPKnwLvLAXw7	Layer2	Fig 7B
Cascade OFF	pGluc-Pgu_crRNA-PspCas13b	https://benchling.com/s/seq-SjvgeWN3ybsNDiRJL3bI	Layer1	Fig 7B
**Description**	**Original name**	**Benchling link**	**Short name**	**Figure**
Cascade ON	pNMD-ON-Gluc-Cj_gRNA-SaCas9	https://benchling.com/s/seq-5I7uNzvDQsiAkve6zy1m?m=slm-lDksahsDX9RECOF60jb2	Mediator	Fig 7D
Cascade ON	pNMD-ON-Gluc-Sa_gRNA-CjCas9	https://benchling.com/s/seq-6Kx7pEc6pNSoW9zg2ss4?m=slm-UIV0kz4BYWfMcJTKSFFq	Mediator	Fig 7D
**Description**	**Original name**	**Benchling link**	**Short name**	**Figure**
Spacer	pSp_gRNA(dSpacer)-EGFP	https://benchling.com/s/seq-lJWs8DgPPdrKACrf9AZG	Sp_gRNA(dSpacer)	Fig S7A
Spacer	pSp_gRNA(dSpacer)_v2-EGFP	https://benchling.com/s/seq-xhy09liqEX93OkdDrZrF	Sp_gRNA(dSpacer)_v2	Fig S7A
Spacer	pSp_gRNA(dSpacer)_v3-EGFP	https://benchling.com/s/seq-Ct1o64lxph3yyjds6eip	Sp_gRNA(dSpacer)_v3	Fig S7A
Spacer	pCj_gRNA(dSpacer)-EGFP	https://benchling.com/s/seq-W5b1eNLM4GA1q1Ps69ys?m=slm-txcdw4VLRMv1n4fn5iTv	Cj_gRNA(dSpacer)	Fig S7B

Spacer	p6nt-Cj_gRNA(dSpacer)-EGFP	https://benchling.com/s/seq-Mb23Fe6xrQ6Vv3OVlaMR?m=slm-4i9Wfy0jpPjhJfeV4MqS	6nt-Cj_gRNA(dSpacer)	Fig S7B
**Description**	**Original name**	**Benchling link**	**Short name**	**Figure**
Tandem	p(Gluc-Psp_crRNA)x2-EGFP	https://benchling.com/s/seq-4BvYpGJYVe1e8L4woMyA?m=slm-jSUXUDwvud4C54EjjOMp	(Gluc-Psp_crRNA)x2	Fig S6A
Tandem	p(Gluc-Pgu_crRNA)x2-EGFP	https://benchling.com/s/seq-RZy20mrb7cVwu1sa8c3x?m=slm-OuYUxb1uDmAxyALld67w	(Gluc-Pgu_crRNA)x2	Fig S6B
Tandem	p(Gluc-Cj_gRNA)x2-EGFP	https://benchling.com/s/seq-Lo7QGsAx2HwDeAOx53P7	(Gluc-Cj_gRNA)x2	Fig S6C
Tandem	p(Gluc-Cj_gRNA)-(Cj_gRNA)-EGFP	https://benchling.com/s/seq-kDcQmJsWuUkvyzF6wOEU	(Gluc-Cj_gRNA)-(Cj_gRNA)	Fig S6C
Tandem	pGluc-Cj_gRNA+30nt-Gluc-Cj_gRNA-EGFP	https://benchling.com/s/seq-ZSZmBhkDtAcJJGj6ZPf9	Cj_gRNA+30nt-Gluc-Cj_gRNA	Fig S6C
**Description**	**Original name**	**Benchling link and Addgene #**	**Short name**	**Figure**
	pcDNA3.1+-myc-HisA	https://benchling.com/s/seq-wZkotHbe8PB30KDVPJqa	Control / No trigger	
	pAptamerCassette-EGFP	Addgene plasmid # 140288	No gRNA / Layer0 (Control)	
	KW1368_pbG-AAVS1-Nst-TO-dCas9-2AmCh	https://benchling.com/s/seq-YxU1T3hiaj1KqWJEVE7b?m=slm-EFMMJ9ZMQ1DFgsWNmr4S	KW1368	

Supplementary Table 6
Primers and template oligo DNA used for generating synthetic mRNAs.

PCR primers

No.	Primer/Oligo DNA name	Sequence (5' → 3')
1	TAP_T7_G3C fwd primer	CAGTGAATTGTAATACGACTCACTATAGGGC
2	IVT_5prime_UTR primer	CAGTGAATTGTAATACGACTCACTATAGGGCGAATTAAGAGAGAAAAGAAGAGTAAGAAGAAAT ATAAGACACCGGTCGCCACCATG
3	Rev5UTR primer	CATGGTGGCGACCGGTGTCTTATATTTCTTCTTACTC
4	IVT_3prime_UTR primer	TCTAGACCTTCTGCGGGGCTTGCCTTCTGGCCATGCCCTTCTTCTCTCCCTTGCACCTGTACCT CTTGGTCTTTGAATAAAGCCTGAGTAGG
5	Fwd3UTR primer	TCTAGACCTTCTGCGGGGC
6	Rev3UTR2T20	TTTTTTTTTTTTTTTTTTTTCCTACTCAGGCTTTATTCAAAGACCAAG
7	SphcCas9 ORF fwd primer	CACCGGTCGCCACCATGGATAAGAAATACAGCATTGGAC
8	SphcCas9 ORF rev primer	GCCCCGCAGAAGGTCTAGACTATCACACCTTCCTCTTCTTCTTGG
9	T7-5UTR for cassette1	TAATACGACTCACTATAggTCAGATCCGCTAGCGGATCC
10	EGFP ORF_Rv	GCCCCGCAGAAGGTCTAGACTACTTGTACAGCTCGTCCATGCCGAGAG
11	YF771_T7_5UTR_fwd	ATTGTAATACGACTCACTATAGGGCGAATTAAGAGAGAAAAGAAGAGTAAG
12	3UTR120A	TTTTTTTTTTTTTTTTTTTTTTTTTTTTTTTTTTTTTTTTTTTTTTTTTTTTTTTTTTTTTTTTTTTTTTTTTTT TTTTTTTTTTTTTTTTTTTTTTTTTTTTTTTTTTTTTTTTTTTTTTCCTACTCAGGCTTTATTCA
13	KWC00434_T7+g_Fw	CAGTGAATTGTAATACGACTCACTATAG
14	KWC944_extension1-3UTR-120A_Rv	TTTTTTTTTTTTTTTTTTTTTTTTTTTTTTTTTTTTTTTTTTTTTTTTTTTTTTTTTTTTTTTTTTTTTTTTTTT TTTTTTTTTTTTTTTTTTTTTTTTTTTTTTTTTTTTTTTTTTTTTTTCCTACTCAGGCTTTATTCaaagaccaag

Key PCR products

No.	Name	Type	Templates/Plasmids	Forward primer	Reverse primer
1	5'-UTR	UTR	IVT_5prime_UTR primer	TAP_T7_G3C fwd primer	Rev5UTR primer
2	3'-UTR	UTR	IVT_3prime_UTR primer	Fwd3UTR primer	Rev3UTR2T20
3	Cas9 mRNA ORF	ORF	pHL-EF1a-SphcCas9-iC-A	SphcCas9 ORF fwd primer	SphcCas9 ORF rev primer
4	Gluc-Sp_gRNA-EGFP ORF	5'UTR-ORF	pGluc-Sp_gRNA-EGFP	T7-5UTR for cassette1	EGFP ORF_Rv
5	EGFP mRNA ORF	ORF-3'UTR	pAptamerCassette-EGFP	EGFP mRNA1 ORF_Fw	EGFP mRNA1 ORF_Rv
6	Cas9 mRNA Template	IVT template	Cas9 mRNA ORF, 5'-UTR, 3'-UTR	TAP_T7_G3C fwd primer	3UTR120A
7	Gluc-Sp_gRNA-EGFP Template	IVT template	Gluc-Sp_gRNA-EGFP ORF, 3'-UTR	T7-5UTR for cassette1	3UTR120A
8	EGFP mRNA Template	IVT template	pUC19-EGFPv2woT7r	YF771_T7_5UTR_fwd	3UTR120A
9	iRFP670 mRNA Template	IVT template	pUC19-iRFP670woT7f	YF771_T7_5UTR_fwd	3UTR120A
10	E3 mRNA Template	IVT template	STKp-66 pUC19-T7E3	KWC00434_T7+g_Fw	KWC944_extension1-3UTR-120A_Rv
11	K3 mRNA Template	IVT template	STKp-67 pUC19-T7K3	KWC00434_T7+g_Fw	KWC944_extension1-3UTR-120A_Rv
12	B18R mRNA Template	IVT template	STKp-68 pUC19-T7B18R ver2	KWC00434_T7+g_Fw	KWC944_extension1-3UTR-120A_Rv

Supplementary Table 7
Transfection tables of all experiments performed in this study.

Figure 1B, E, Figure 2, Figure S2, S3A, S6, S7, S8, S10 (24-well plate)

Switch plasmid	100 ng
Trigger plasmid	400 ng
Reference plasmid	100 ng
Opti-MEM	up to 100 μL
Lipofectamine 2000	2 μL

Figure 1C, Figure S4 (24-well plate): HEK293FT, iPSC

Switch/control mRNA	100 ng
Trigger mRNA	100 ng
Reference mRNA	100 ng
Opti-MEM	up to 50 μL
Lipofectamine MessengerMax	1 μL

Figure 1C, Figure S4 (24-well plate): HeLa, A549

Switch/control mRNA	100 ng
Trigger mRNA	100 ng
E3 mRNA	40 ng
K3 mRNA	40 ng
B18R mRNA	40 ng
Reference mRNA	100 ng
Opti-MEM	up to 50 μL
Lipofectamine MessengerMax	1 μL

Figure 1D, Figure S5 (24-well plate)

Switch mRNA	100 ng
Reference mRNA	100 ng
Opti-MEM	up to 50 μL
Lipofectamine MessengerMax	1 μL
Doxycycline (Final: 1 μg/mL)	(+) or (-)

Figure 3B (24-well plate)

	[N-Cas9, C-Cas9]<br>[-,-]	[N-Cas9, C-Cas9]<br>[+,-]	[N-Cas9, C-Cas9]<br>[-,+]	[N-Cas9, C-Cas9]<br>[+,+]	WT
pGluc-Sp_gRNA-EGFP	100 ng	100 ng	100 ng	100 ng	100 ng
pcDNA3.1-myc-His6	800 ng	400 ng	400 ng		400 ng
pcDNA3.1-SpCas9					400 ng
pcDNA3.1-SpCas9(1-713)-N_intein		400 ng		400 ng	
pcDNA3.1-C_intein-SpCas9(714-1368)			400 ng	400 ng	
pCMV-tdiRFP670	100 ng	100 ng	100 ng	100 ng	100 ng

Figure 3D (24-well plate)

	A/C heterodimerizer (-)					
	WT		Split		No trigger	
	No_gRNA	Sp_gRNA	No_gRNA	Sp_gRNA	No_gRNA	Sp_gRNA
pAptamerCassette-EGFP	100 ng		100 ng		100 ng	
pGluc-Sp_gRNA-EGFP		100 ng		100 ng		100 ng
pcDNA3.1-SpCas9(1-713)-(GGGGS)3-DmrA			400 ng	400 ng		
pcDNA3.1-DmrC-(GGGGS)3-SpCas9(714-1368)			400 ng	400 ng		
pcDNA3.1-myc-His6	400 ng	400 ng			800 ng	800 ng
pcDNA3.1-SpCas9	400 ng	400 ng				
	A/C heterodimerizer (+)					
	WT		Split		No trigger	
	No_gRNA	Sp_gRNA	No_gRNA	Sp_gRNA	No_gRNA	Sp_gRNA
pAptamerCassette-EGFP	100 ng		100 ng		100 ng	
pGluc-Sp_gRNA-EGFP		100 ng		100 ng		100 ng
pcDNA3.1-SpCas9(1-713)-(GGGGS)3-DmrA			400 ng	400 ng		
pcDNA3.1-DmrC-(GGGGS)3-SpCas9(714-1368)			400 ng	400 ng		
pcDNA3.1-myc-His6	400 ng	400 ng			800 ng	800 ng
pcDNA3.1-SpCas9	400 ng	400 ng				

Figure 3F (24-well plate)

	0 ng	200 ng	400 ng	1000 ng	2000 ng
pGluc-Sa_gRNA-EGFP<br>or<br>pAptamerCassette-EGFP	100 ng	100 ng	100 ng	100 ng	100 ng
pcDNA3.1-SaCas9	200 ng	200 ng	200 ng	200 ng	200 ng
pcDNA3.1-myc-His6	2000 ng	1000 ng	400 ng	200 ng	0 ng
pcDNA3.1+-AcrIIC2_Nm	0 ng	200 ng	400 ng	1000 ng	2000 ng
pCMV-tdiRFP670	100 ng	100 ng	100 ng	100 ng	100 ng

Figure 4, Figure S13, S14 (384-well plate)

Switch plasmid	31.25 ng
Trigger plasmid	125 ng
Reference plasmid	31.25 ng
Opti-MEM	up to 10 μL
Lipofectamine 2000	0.4 μL

Figure 5 (24-well plate)

notation	plasmid name	
dSpCas9-VPR	Sp_dCas9-VPR<br>or<br>pcDNA3.1+-myc-HisA	400 ng
gRNA	pHL-gRNA[TRE new]-iRFP-RIH<br>or<br>pHL-Sp_lgRNA_Nluc-iRFP-RIH	100 ng
pTRE-hmAG1	pTRE-Tight-hmAG1	100 ng
pSp_gRNA-RFP	pGluc-Sp_gRNA-tagRFP<br>or<br>pcDNA3.1+-myc-HisA	100 ng
reference	pAptamerCassette-tagBFP	100 ng

Figure 6B (96-well plate)

Trigger plasmid 1	200 ng
Trigger plasmid 2	200 ng
Mediator plasmid 1	50 ng
Mediator plasmid 2	50 ng
Reporter plasmid	12.5 ng
Reference plasmid	25 ng
Opti-MEM	up to 20 μL
Lipofectamine 2000	0.5 μL

<table>
<tr><th>Figure 6F, Figure S18 (24-well plate): Pgu, Sa, Psp</th><th>[0,0]</th><th>[0,1]</th><th>[1,0]</th><th>[1,1]</th><th>hBax only</th><th>Empty</th><th>Mock</th></tr>
<tr><td>pcDNA3.1+-myc-HisA</td><td>1700 ng</td><td>900 ng</td><td>900 ng</td><td>100 ng</td><td>2100 ng</td><td>2150 ng</td><td>0 ng</td></tr>
<tr><td>pcDNA3.1+-PguCas13b-NES</td><td>0 ng</td><td>800 ng</td><td>0 ng</td><td>800 ng</td><td>0 ng</td><td>0 ng</td><td>0 ng</td></tr>
<tr><td>pcDNA3.1+-SaCas9</td><td>0 ng</td><td>0 ng</td><td>800 ng</td><td>800 ng</td><td>0 ng</td><td>0 ng</td><td>0 ng</td></tr>
<tr><td>pGluc-Pgu_crRNA-PspCas13b</td><td colspan="4">200 ng</td><td>0 ng</td><td>0 ng</td><td>0 ng</td></tr>
<tr><td>pGluc-Sa_gRNA-PspCas13b-NES</td><td colspan="4">200 ng</td><td>0 ng</td><td>0 ng</td><td>0 ng</td></tr>
<tr><td>pGluc-Psp_crRNA-hBax</td><td colspan="5">50 ng</td><td>0 ng</td><td>0 ng</td></tr>
<tr><td>Opti-MEM</td><td colspan="7">Up to 100 μL</td></tr>
<tr><td>Lipofectamine 2000</td><td colspan="7">2 μL</td></tr>
</table>

<table>
<tr><th>Figure 6F, Figure S18 (24-well plate): Psp, Sa, Ak</th><th>[0,0]</th><th>[0,1]</th><th>[1,0]</th><th>[1,1]</th><th>hBax only</th><th>Empty</th><th>Mock</th></tr>
<tr><td>pcDNA3.1+-myc-HisA</td><td>1700 ng</td><td>900 ng</td><td>900 ng</td><td>100 ng</td><td>2100 ng</td><td>2150 ng</td><td>0 ng</td></tr>
<tr><td>pcDNA3.1-PspCas13b(WT)-NES-myc-His6</td><td>0 ng</td><td>800 ng</td><td>0 ng</td><td>800 ng</td><td>0 ng</td><td>0 ng</td><td>0 ng</td></tr>
<tr><td>pcDNA3.1+-SaCas9</td><td>0 ng</td><td>0 ng</td><td>800 ng</td><td>800 ng</td><td>0 ng</td><td>0 ng</td><td>0 ng</td></tr>
<tr><td>pGluc-Psp_crRNA-AkCas12b</td><td colspan="4">200 ng</td><td>0 ng</td><td>0 ng</td><td>0 ng</td></tr>
<tr><td>pGluc-Sa_gRNA-AkCas12b</td><td colspan="4">200 ng</td><td>0 ng</td><td>0 ng</td><td>0 ng</td></tr>
<tr><td>pAkCas12b_sgRNA_v1-hBax</td><td colspan="5">50 ng</td><td>0 ng</td><td>0 ng</td></tr>
<tr><td>Opti-MEM</td><td colspan="7">Up to 100 μL</td></tr>
<tr><td>Lipofectamine 2000</td><td colspan="7">2 μL</td></tr>
</table>

Figure 7B (24-well plate)

Switch/control plasmid	25 ng
Layer 1	50 ng
Layer 2	50 ng
Layer 3	200 ng
Layer 4	800 ng
Opti-MEM	up to 100 μL
Lipofectamine 2000	2 μL

Figure 7D (24-well plate)

Trigger plasmid	400 ng
Mediator plasmid	200 ng
ON switch plasmid	100 ng
Reference plasmid	100 ng
Opti-MEM	up to 100 μL
Lipofectamine 2000	2 μL

Figure 7F (24-well plate)

Trigger plasmid 1	400 ng
Trigger plasmid 2	400 ng
NOR switch plasmid	100 ng
Reference plasmid	100 ng
Opti-MEM	up to 100 μL
Lipofectamine 2000	2 μL

Figure 8 (24-well plate)	[0,0]	[0,1]	[1,0]	[1,1]
pTRE-Gluc_Sa_gRNA-hmA1	400 ng			
pHL-Sp_lgRNA_a-iRFP-RIH	100 ng			
pHL-Sa_lgRNA_a-iRFP-RIH	100 ng			
pcDNA3.1-myc-HisA	800 ng	400 ng	400 ng	0 ng
pcDNA3.1-dSpCas9-VPR	0 ng	400 ng	0 ng	400 ng
pcDNA3.1-dSaCas9-VPR	0 ng	0 ng	400 ng	400 ng
pSa_lgRNA_a-CMVmin-Gluc_Sp_gRNA-tagBFP	100 ng			
pSp_lgRNA_ax2-CMVmin-Gluc_Sa_gRNA-tagBFP-Tri-HHR-Sp_gRNA[TRE]-HDVR	400 ng			
Opti-MEM	Up to 100 μL			
Lipofectamine 2000	2 μL			

Figure S3B (24-well plate)

Switch/control plasmid	100 ng
Trigger plasmid	100 ng
Acr (AcrIIA4) plasmid	100 ng
Reference plasmid	100 ng
Opti-MEM	up to 20 μL
Lipofectamine 2000	0.5 μL

Figure S12 (24-well plate)

	[N-Cas9, C-Cas9] [-,-]	[N-Cas9, C-Cas9] [+,-]	[N-Cas9, C-Cas9] [-,+]	[N-Cas9, C-Cas9] [+,+]	WT
pGluc-Sp_gRNA-EGFP	100 ng	100 ng	100 ng	100 ng	100 ng
pcDNA3.1-myc-His6	800 ng	400 ng	400 ng		400 ng
pcDNA3.1-SpCas9					400 ng
pcDNA3.1-SpCas9(1-713)		400 ng		400 ng	
pcDNA3.1-SpCas9(714-1368)			400 ng	400 ng	
pCMV-tdiRFP670	100 ng	100 ng	100 ng	100 ng	100 ng

Figure S17 (24-well plate)

notation	plasmid name	
OFF switch	pGluc-Sa_gRNA-tagRFP<br>or<br>pAptamerCassette-tagRFP2	100 ng
ON switch	pNMD-ON-Gluc-Sa_gRNA<br>or<br>pNMD-ON-Gluc-Sa_gRNA	100 ng
trigger	pcDNA3.1 + -SaCas9<br>or<br>pcDNA3.1 + -myc-HisA	400 ng
reference	pCMV-tdiRFP670	100 ng

Supplementary Sequences

RNA sequences used in this study.

1. The 5' termini of mRNA were capped with ARCA.
2. The protein coding regions are shown as bold letters.
3. The start and stop codons are underlined.
4. The sequence of Gluc-Sp_gRNA is shown in underlined italic letters.

Cas9 mRNA

GGGCGAAUUAAGAGAGAAAAGAAGAGUAAGAAGAAAUAUAAGACACCGGUCGCCACC**AUGGAUAAGAAAUAC**
AGCAUUGGACUGGACAUUGGGACAAACUCCGUGGGAUGGGCCGUGAUUACAGACGAAUACAAAGUGCCUU
CAAAGAAGUUCAAGGUGCUGGGCAACACCGAUAGACACAGCAUCAAGAAAAAUCUGAUUGGAGCCCUGCUG
UUCGACUCCGGCGAGACAGCUGAAGCAACUCGGCUGAAAAGAACUGCUCGGAGAAGGUAUACCCGCCGAA
AGAAUAGGAUCUGCUACCUGCAGGAGAUUUUCAGCAACGAAAUGGCCAAGGUGGACGAUAGUUUCUUUCAC
CGCCUGGAGGAAUCAUUCCUGGUCGAGGAAGAUAAGAAACACGAGCGGCAUCCCAUCUUUGGCAACAUUG
UGGACGAGGUCGCUUAUCACGAAAAGUACCCUACCAUCUAUCAUCUGAGGAAGAAACUGGUGGACUCCACA
GAUAAAGCAGACCUGCGCCUGAUCUAUCUGGCCCUGGCUCACAUGAUUAAGUUCCGGGGCCAUUUUCUGAU
CGAGGGGGAUCUGAACCCAGACAAUUCUGAUGUGGACAAGCUGUUCAUCCAGCUGGUCCAGACAUACAAUC
AGCUGUUUGAGGAAAACCCCAUUAAUGCAUCUGGCGUGGACGCAAAAGCCAUCCUGAGUGCCAGACUGUCU
AAGAGUCGGAGACUGGAGAACCUGAUCGCUCAGCUGCCAGGGGAAAAGAAAAACGGCCUGUUUGGGAAUC
UGAUUGCACUGUCACUGGGACUGACUCCCAACUUCAAGAGCAAUUUUGAUCUGGCCGAGGACGCUAAACUG
CAGCUGUCCAAGGACACCUAUGACGAUGACCUGGAUAACCUGCUGGCUCAGAUCGGGGAUCAGUACGCAG
ACCUGUUCCUGGCCGCUAAGAAUCUGUCUGACGCCAUCCUGCUGAGUGAUAUUCUGCGCGUGAACACCGAG
AUUACAAAAGCCCCCCUGUCAGCUAGCAUGAUCAAGAGAUAUGACGAGCACCAUCAGGAUCUGACCCUGCU
GAAGGCUCUGGUGAGGCAGCAGCUGCCUGAGAAGUACAAGGAAAUCUUCUUUGAUCAGUCUAAGAACGGA
UACGCCGGCUAUAUUGACGGCGGGGCUAGUCAGGAGGAGUUCUACAAGUUUAUCAAACCCAUUCUGGAGA
AGAUGGAUGGCACAGAGGAACUGCUGGUGAAACUGAAUCGGGAAGACCUGCUGAGGAAGCAGCGCACUUU
UGAUAACGGAAGCAUCCCUCACCAGAUUCAUCUGGGAGAGCUGCACGCAAUCCUGAGGCGCCAGGAAGAC
UUCUACCCAUUUCUGAAGGAUAACAGGGAGAAGAUCGAAAAAAUUCUGACAUUCCGCAUCCCCUACUAUGU
GGGCCCUCUGGCAAGAGGCAACAGCCGGUUUGCCUGGAUGACUCGCAAAUCUGAGGAAACAAUCACUCCC
UGGAACUUCGAGGAAGUGGUCGAUAAGGGCGCUUCCGCACAGUCUUUCAUUGAGCGGAUGACAAACUUCG
ACAAGAACCUGCCAAACGAAAAAGUGCUGCCCAAGCACUCUCUGCUGUACGAGUAUUUCACAGUCUAUAAC
GAACUGACUAAGGUGAAAUACGUCACCGAGGGGAUGAGAAAGCCUGCCUUCCUGAGUGGAGAACAGAAGA
AAGCUAUCGUGGACCUGCUGUUUAAAACCAAUAGGAAGGUGACAGUCAAGCAGCUGAAAGAGGACUAUUUC
AAGAAAAUUGAAUGUUUCGAUUCUGUGGAGAUCAGUGGCGUCGAAGACAGGUUUAACGCCUCCCUGGGGA
CCUACCACGAUCUGCUGAAGAUCAUUAAGGAUAAAGACUUCCUGGACAACGAGGAAAAUGAGGAUAUCCUG
GAAGACAUUGUGCUGACCCUGACACUGUUUGAGGAUAGGGAAAUGAUCGAGGAACGCCUGAAGACCUAUG
CCCAUCUGUUCGAUGACAAAGUGAUGAAACAGCUGAAGCGACGGAGAUACACAGGAUGGGGCCGACUGUC
UCGGAAGCUGAUCAAUGGGAUUCGCGACAAACAGAGUGGAAAGACCAUCCUGGACUUUCUGAAAUCAGAU
GGCUUCGCCAACCGGAACUUCAUGCAGCUGAUUCACGAUGACAGCCUGACAUUCAAAGAGGAUAUCCAGAA
GGCACAGGUGUCCGGGCAGGGAGACUCUCUGCACGAGCAUAUCGCAAACCUGGCCGGCAGCCCUGCCAUC
AAGAAAGGGAUUCUGCAGACCGUGAAGGUGGUGGACGAGCUGGUGAAAGUCAUGGGAAGACAUAAGCCAG
AAAACAUCGUGAUUGAGAUGGCCAGGGAAAAUCAGACCACACAGAAAGGCCAGAAGAACUCAAGGGAGCG
CAUGAAAAGAAUCGAGGAAGGAAUUAAGGAACUGGGCAGCCAGAUCCUGAAAGAGCACCCCGUGGAAAAC
ACACAGCUGCAGAAUGAGAAGCUGUAUCUGUACUAUCUGCAGAAUGGACGCGAUAUGUACGUGGACCAGG
AGCUGGAUAUUAACCGACUGUCCGAUUACGACGUGGAUCAUAUCGUCCCACAGUCAUUCCUGAAAGAUGAC
AGCAUUGACAAUAAGGUGCUGACCCGCUCUGACAAAAACCGAGGCAAGAGUGAUAAUGUCCCCUCAGAGGA
AGUGGUCAAGAAAAUGAAGAACUACUGGAGGCAGCUGCUGAAUGCCAAACUGAUCACACAGCGAAAGUUU
GAUAACCUGACUAAAGCUGAGCGGGGAGGCCUGAGUGAACUGGACAAAGCAGGCUUCAUUAAGCGACAGC
UGGUGGAGACACGGCAGAUCACAAAGCACGUCGCCCAGAUUCUGGAUUCAAGAAUGAACACUAAGUACGAU
GAGAAUGACAAACUGAUCAGAGAAGUGAAGGUCAUUACCCUGAAGUCAAAACUGGUGAGCGACUUUCGGA
AAGAUUUCCAGUUUUAUAAGGUCAGAGAGAUCAACAACUACCACCAUGCUCAUGACGCAUACCUGAACGCA
GUGGUCGGCACAGCCCUGAUUAAGAAAUACCCUAAACUGGAGUCCGAGUUCGUGUACGGGGACUAUAAGG
UGUACGAUGUCAGAAAAAUGAUCGCCAAGUCUGAGCAGGAAAUUGGCAAAGCCACUGCUAAGUAUUUCUUU
UACAGUAACAUCAUGAAUUUCUUUAAGACUGAGAUCACCCUGGCAAAUGGGGAAAUCCGAAAGCGGCCACU
GAUUGAGACUAACGGCGAGACAGGAGAAAUCGUGUGGGACAAAGGAAGAGAUUUUGCUACCGUGAGGAAG
GUCCUGAGCAUGCCCCAAGUGAAUAUUGUCAAGAAAACAGAGGUGCAGACUGGGGGAUUCAGUAAGGAAU
CAAUUCUGCCUAAACGCAACUCCGAUAAGCUGAUCGCCCGAAAGAAAGACUGGGACCCCAAGAAGUAUGGC
GGGUUCGACUCCCCAACUGUGGCUUACUCUGUCCUGGUGGUCGCAAAGGUGGAGAAGGGAAAAAGCAAGA
AACUGAAAUCCGUCAAGGAACUGCUGGGCAUCACCAUUAUGGAGCGCAGCUCCUUCGAAAAGAAUCCUAUC
GAUUUUCUGGAGGCCAAAGGCUAUAAGGAAGUGAAGAAAGACCUGAUCAUCAAGCUGCCAAAGUACUCACU
GUUUGAGCUGGAAAACGGGAGAAAGAGGAUGCUGGCAAGCGCCGGGGAGCUGCAGAAAGGAAAUGAACUG
GCCCUGCCCUCCAAGUACGUGAACUUCCUGUAUCUGGCUAGCCACUACGAGAAGCUGAAAGGGUCCCCUGA
GGAUAACGAACAGAAACAGCUGUUUGUGGAGCAGCACAAGCAUUAUCUGGACGAGAUCAUUGAACAGAUUA

GCGAGUUCUCCAAAAGAGUGAUCCUGGCUGACGCAAAUCUGGAUAAGGUCCUGAGCGCAUACAACAAACAC
CGGGAUAAGCCAAUCAGAGAGCAGGCCGAAAAUAUCAUUCAUCUGUUCACUCUGACCAACCUGGGAGCCCC
CGCAGCCUUCAAGUAUUUUGACACUACCAUCGAUCGCAAACGAUACACAAGCACUAAGGAGGUGCUGGACG
CUACCCUGAUUCAUCAGAGCAUUACUGGCCUGUAUGAAACAAGGAUUGACCUGUCUCAGCUGGGCGGCGAC
UCCGGAGCUGACCCCAAGAAGAAGAGGAAGGUG<u>UGA</u>UAGUCUAGACCUUCUGCGGGGCUUGCCUUCUGGC
CAUGCCCUUCUUCUCUCCCUUGCACCUGUACCUCUUGGUCUUUGAAUAAAGCCUGAGUAGGAAAAAAAAAAAA
AAAAAAAAAAAAAAAAAAAAAAAAAAAAAAAAAAAAAAAAAAAAAAAAAAAAAAAAAAAAAAAAAAAAAAAAAAAAAAA
AAAAAAAAAAAAAAAAAAAAAAAAAAAAAA

Gluc-Sp_gRNA-EGFP
GGUCAGAUCCGCUAGCGGAUCC<u>*GAGAUCAGGGCAAACAGAACUGUUUUAGAGCUAGAAAUAGCAAGUUAAAA*</u>
<u>*UAAGGCUAGUCCGUUAUCAACUUGAAAAAGUGGCACCGAGUCGGUGC*</u>AGAUCUACCGGUCGCCACC**<u>AUG</u>GUG**
AGCAAGGGCGAGGAGCUGUUCACCGGGGUGGUGCCCAUCCUGGUCGAGCUGGACGGCGACGUAAACGGCC
ACAAGUUCAGCGUGUCCGGCGAGGGCGAGGGCGAUGCCACCUACGGCAAGCUGACCCUGAAGUUCAUCUG
CACCACCGGCAAGCUGCCCGUGCCCUGGCCCACCCUCGUGACCACCCUGACCUACGGCGUGCAGUGCUUCA
GCCGCUACCCCGACCACAUGAAGCAGCACGACUUCUUCAAGUCCGCCAUGCCCGAAGGCUACGUCCAGGAG
CGCACCAUCUUCUUCAAGGACGACGGCAACUACAAGACCCGCGCCGAGGUGAAGUUCGAGGGCGACACCC
UGGUGAACCGCAUCGAGCUGAAGGGCAUCGACUUCAAGGAGGACGGCAACAUCCUGGGGCACAAGCUGGA
GUACAACUACAACAGCCACAACGUCUAUAUCAUGGCCGACAAGCAGAAGAACGGCAUCAAGGUGAACUUCA
AGAUCCGCCACAACAUCGAGGACGGCAGCGUGCAGCUCGCCGACCACUACCAGCAGAACACCCCCAUCGGC
GACGGCCCCGUGCUGCUGCCCGACAACCACUACCUGAGCACCCAGUCCGCCCUGAGCAAAGACCCCAACGA
GAAGCGCGAUCACAUGGUCCUGCUGGAGUUCGUGACCGCCGCCGGGAUCACUCUCGGCAUGGACGAGCUG
UACAAG<u>UAG</u>UCUAGACCUUCUGCGGGGCUUGCCUUCUGGCCAUGCCCUUCUUCUCUCCCUUGCACCUGUAC
CUCUUGGUCUUUGAAUAAAGCCUGAGUAGGAAAAAAAAAAAAAAAAAAAAAAAAAAAAAAAAAAAAAAAAAAAAAAAAAA
AAAAAAAAAAAAAAAAAAAAAAAAAAAAAAAAAAAAAAAAAAAAAAAAAAAAAAAAAAAAAAAAAAAAAAAAAAAA

EGFP mRNA
GGCCGCUUGAAGUCUUUAAUUAAACCCGCUUGAAGUCUUUAAUUAAACGAACGGGCACGCUGACAAUUCAAG
CACUCUGAUUUGACAAUUACAAGCACUCUGAUUUGACAAUUACACCGGUCGCCACC**<u>AUG</u>GUGAGCAAGGGCG**
AGGAGCUGUUCACCGGGGUGGUGCCCAUCCUGGUCGAGCUGGACGGCGACGUAAACGGCCACAAGUUCAG
CGUGUCCGGCGAGGGCGAGGGCGAUGCCACCUACGGCAAGCUGACCCUGAAGUUCAUCUGCACCACCGGC
AAGCUGCCCGUGCCCUGGCCCACCCUCGUGACCACCCUGACCUACGGCGUGCAGUGCUUCAGCCGCUACCC
CGACCACAUGAAGCAGCACGACUUCUUCAAGUCCGCCAUGCCCGAAGGCUACGUCCAGGAGCGCACCAUCU
UCUUCAAGGACGACGGCAACUACAAGACCCGCGCCGAGGUGAAGUUCGAGGGCGACACCCUGGUGAACCG
CAUCGAGCUGAAGGGCAUCGACUUCAAGGAGGACGGCAACAUCCUGGGGCACAAGCUGGAGUACAACUAC
AACAGCCACAACGUCUAUAUCAUGGCCGACAAGCAGAAGAACGGCAUCAAGGUGAACUUCAAGAUCCGCCA
CAACAUCGAGGACGGCAGCGUGCAGCUCGCCGACCACUACCAGCAGAACACCCCCAUCGGCGACGGCCCC
GUGCUGCUGCCCGACAACCACUACCUGAGCACCCAGUCCGCCCUGAGCAAAGACCCCAACGAGAAGCGCGA
UCACAUGGUCCUGCUGGAGUUCGUGACCGCCGCCGGGAUCACUCUCGGCAUGGACGAGCUGUACAAG<u>UAA</u>
AGCGGCCGCGACUCUAGAUCAUAAUCAGCCAUACCACAUUUGUAGAGGUUUUACUUGCUUUAAAAAACCUCCC
ACACCUCCCCCUGAACCUGAAACAUAAAAUGAAUGCAAUUGUUGUUGUUAACUUGUUUAUUGCAGCUUAUAAU
GGUUACAAAUAAAGCAAUAGCAUCACAAAUUUCACAUGAAUAAAGCCUGAGUAGGAAAAAAAAAAAAAAAAAAAA
AAAAAAAAAAAAAAAAAAAAAAAAAAAAAAAAAAAAAAAAAAAAAAAAAAAAAAAAAAAAAAAAAAAAAAAAAAAAAAA
AAAAAAAAAAAAAAAAAAAAAA

iRFP670 mRNA
GGGCGAAUUAAGAGAGAAAAGAAGAGUAAGAAGAAAUAUAAGACACCGGUCGCCACC**<u>AUG</u>GCGCGUAAGGUC**
GAUCUCACCUCCUGCGAUCGCGAGCCGAUCCACAUCCCCGGCAGCAUUCAGCCGUGCGGCUGCCUGCUAGC
CUGCGACGCGCAGGCGGUGCGGAUCACGCGCAUUACGGAAAAUGCCGGCGCGUUCUUUGGACGCGAAACU
CCGCGGGUCGGUGAGCUACUCGCCGAUUACUUCGGCGAGACCGAAGCCCAUGCGCUGCGCAACGCACUGG
CGCAGUCCUCCGAUCCAAAGCGACCGGCGCUGAUCUUCGGUUGGCGCGACGGCCUGACCGGCCGCACCUU
CGACAUCUCACUGCAUCGCCAUGACGGUACAUCGAUCAUCGAGUUCGAGCCUGCGGCGGCCGAACAGGCC
GACAAUCCGCUGCGGCUGACGCGGCAGAUCAUCGCGCGCACCAAAGAACUGAAGUCGCUCGAAGAGAUGG
CCGCACGGGUGCCGCGCUAUCUGCAGGCGAUGCUCGGCUAUCACCGCGUGAUGUUGUACCGCUUCGCGGA
CGACGGCUCCGGGAUGGUGAUCGGCGAGGCGAAGCGCAGCGACCUCGAGAGCUUUCUCGGUCAGCACUUU
CCGGCGUCGCUGGUCCCGCAGCAGGCGCGGCUACUGUACUUGAAGAACGCGAUCCGCGUGGUCUCGGAUU
CGCGCGGCAUCAGCAGCCGGAUCGUGCCCGAGCACGACGCCUCCGGCGCCGCGCUCGAUCUGUCGUUCGC
GCACCUGCGCAGCAUCUCGCCCUGCCAUCUCGAAUUUCUGCGGAACAUGGGCGUCAGCGCCUCGAUGUCG
CUGUCGAUCAUCAUUGACGGCACGCUAUGGGGAUUGAUCAUCUGUCAUCAUUACGAGCCGCGUGCCGUGC
CGAUGGCGCAGCGCGUCGCGGCCGAAAUGUUCGCCGACUUCUUAUCGCUGCACUUCACCGCCGCCCACCAC
CAACGCAGAUCUCAUAUGCAUCUCGAG<u>UGA</u>UAGUCUAGACCUUCUGCGGGGCUUGCCUUCUGGCCAUGCCC
UUCUUCUCUCCCUUGCACCUGUACCUCUUGGUCUUUGAAUAAAGCCUGAGUAGGAAAAAAAAAAAAAAAAAAA
AAAAAAAAAAAAAAAAAAAAAAAAAAAAAAAAAAAAAAAAAAAAAAAAAAAAAAAAAAAAAAAAAAAAAAAAAAAAAAA
AAAAAAAAAAAAAAAAAAAAAAA

E3 mRNA
GGGCGAAUUAAGAGAGAAAAGAAGAGUAAGAAAUAUAAGACACCGGUCGCCACC**<u>AUG</u>UCUAAAAUCUAUA**
UCGACGAGCGUUCUAACGCAGAGAUUGUGUGAGGCUAUUAAAACCAUUGGAAUCGAAGGAGCUACUGC
UGCACAACUAACUAGACAACUUAAUAUGGAGAAGCGAGAAGUUAAUAAAGCUCUGUACGAUCUUCAACGUA
GUGCUAUGGUGUACAGCUCCGACGAUAUUCCUCCUCGUUGGUUUAUGACAACGGAGGCGGAUAAGCCGGA
UGCUGAUGCUAUGGCUGACGUCAUAAUAGAUGAUGUAUCCCGCGAAAAAUCAAUGAGAGAGGAUCAUAAGU
CUUUUGAUGAUGUUAUUCCGGCUAAAAAAAUUAUUGAUUGGAAAGGUGCUAACCCUGUCACCGUUAUUAAU
GAGUACUGCCAAAUUACUAGGAGAGAUUGGUCUUUUCGUAUUGAAUCAGUGGGGCCUAGUAACUCUCCUAC
AUUUUAUGCCUGUGUAGACAUCGACGGAAGAGUAUUCGAUAAGGCAGAUGGAAAAUCUAAACGAGAUGCUA
AAAAUAAUGCAGCUAAAUUGGCAGUAGAUAAACUUCUUGGUUACGUCAUCAUUAGAUUCGAUUACAAGGAU
GACGACGAUAAG<u>UGA</u>UCUAGACCUUCUGCGGGGCUUGCCUUCUGGCCAUGCCCUUCUUCUCUCCCUUGCAC
CUGUACCUCUUGGUCUUUGAAUAAAGCCUGAGUAGGAAAAAAAAAAAAAAAAAAAAAAAAAAAAAAAAAAAAAAAA
AAAAAAAAAAAAAAAAAAAAAAAAAAAAAAAAAAAAAAAAAAAAAAAAAAAAAAAAAAAAAAAAAAAAAAAAAAAAAA
AAA

K3 mRNA
GGGCGAAUUAAGAGAGAAAAGAAGAGUAAGAAAUAUAAGACACCGGUCGCCACC**<u>AUG</u>CUGGCGUUUUGU**
UACAGCCUUCCAAAUGCAGGAGACGUGAUAAAAGGAAGAGUCUAUGAGAAGGAUUAUGCACUCUAUAUUUA
CCUGUUUGAUUAUCCCCAUUUUGAAGCAAUACUUGCCGAGAGCGUGAAGAUGCGGAUGGAUCGGUAUGUC
GAAUACCGCGACAAGCUUGUAGGGAAAACUGUCAAGGUAAAGGUCAUCAGGGUAGACUAUACCAAAGGAU
AUAUUGAUGUCAACUAUAAGCGCAUGUGUAGGCAUCAAGAUUACAAGGAUGACGACGAUAAG<u>UGA</u>UCUAGA
CCUUCUGCGGGGCUUGCCUUCUGGCCAUGCCCUUCUUCUCUCCCUUGCACCUGUACCUCUUGGUCUUUGAA
UAAAGCCUGAGUAGGAAAAAAAAAAAAAAAAAAAAAAAAAAAAAAAAAAAAAAAAAAAAAAAAAAAAAAAAAAAA
AAAAAAAAAAAAAAAAAAAAAAAAAAAAAAAAAAAAAAAAAAAAAAAAAAAAAAAAAA

B18R mRNA
GGGCGAAUUAAGAGAGAAAAGAAGAGUAAGAAAUAUAAGACACCGGUCGCCACC**<u>AUG</u>GGCACGAUGAAA**
AUGAUGGUACAUAUAUAUUUCGUAUCAUUAUUGUUAUUGCUAUUCCACAGUUACGCCAUAGACAUCGAAAA
UGAAAUCACAGAAUUCUUCAAUAAAAUGAGAGAUACUCUACCAGCUAAAGACUCUAAAUGGUUGAAUCCAG
CAUGUAUGUUCGGAGGCACAAUGAAUGAUAUAGCCGCUCUAGGAGAGCCAUUCAGCGCAAAGUGUCCUCCU
AUUGAAGACAGUCUUUUAUCGCACAGAUAUAAAGACUAUGUGGUUAAAUGGGAAAGGCUAGAAAAAAAUAG
ACGGCGACAGGUUUCUAAUAAACGUGUUAAACAUGGUGAUUUAUGGAUAGCCAACUAUACAUCUAAAUUCA
GUAACCGUAGGUAUUUGUGCACCGUAACUACAAAGAAUGGUGACUGUGUUCAGGGUAUAGUUAGAUCUCAU
AUUAGAAAACCUCCUUCAUGCAUUCCAAAAACAUAUGAACUAGGUACUCAUGAUAAGUAUGGCAUAGACUU
AUACUGUGGAAUUCUUUACGCAAAACAUUAUAAUAAUAUAACUUGGUAUAAAGAUAAUAAGGAAAUUAAUA
UCGACGACAUUAAGUAUUCACAAACGGGAAAGGAAUUAAUUAUUCAUAAUCCAGAGUUAGAAGAUAGCGGA
AGAUACGACUGUUACGUUCAUUACGACGACGUUAGAAUCAAGAAUGAUAUCGUAGUAUCAAGAUGUAAAAU
ACUUACGGUUAUACCGUCACAAGACCACAGGUUUAAACUAAUACUAGAUCCAAAAAUCAACGUAACGAUAG
GAGAACCUGCCAAUAUAACAUGCACUGCUGUGUCAACGUCAUUAUUGAUUGACGAUGUACUGAUUGAAUGG
GAAAAUCCAUCCGGAUGGCUUAUAGGAUUCGAUUUUGAUGUAUACUCUGUUUUAACUAGUAGAGGCGGUAU
UACCGAGGCGACCUUGUACUUUGAAAAUGUUACUGAAGAAUAUAUAGGUAAUACAUAUAAAUGUCGUGGAC
ACAACUAUUAUUUUGAAAAAACCCUUACAACUACAGUAGUAUUGGAGGAUUACAAGGAUGACGACGAUAAG
<u>UGA</u>UCUAGACCUUCUGCGGGGCUUGCCUUCUGGCCAUGCCCUUCUUCUCUCCCUUGCACCUGUACCUCUUG
GUCUUUGAAUAAAGCCUGAGUAGGAAAAAAAAAAAAAAAAAAAAAAAAAAAAAAAAAAAAAAAAAAAAAAAAAAAA
AAAAAAAAAAAAAAAAAAAAAAAAAAAAAAAAAAAAAAAAAAAAAAAAAAAAAAAAAAAAAAAAAAAAAAAA

Supplementary References

1. Esvelt, K. M. *et al.* Orthogonal Cas9 proteins for RNA-guided gene regulation and editing. *Nat Methods* **10**, 1116–1123 (2013).
2. Endo, K., Stapleton, J. A., Hayashi, K., Saito, H. & Inoue, T. Quantitative and simultaneous translational control of distinct mammalian mRNAs. *Nucleic Acids Res* **41**, 1–12 (2013).
3. Yamada, M. *et al.* Crystal Structure of the Minimal Cas9 from Campylobacter jejuni Reveals the Molecular Diversity in the CRISPR-Cas9 Systems. *Mol Cell* **65**, 1109-1121.e3 (2017).
4. Mali, P. *et al.* RNA-Guided Human Genome Engineering via Cas9. *Science* **339**, 823–826 (2013).
5. Li, H. L. *et al.* Precise correction of the dystrophin gene in duchenne muscular dystrophy patient induced pluripotent stem cells by TALEN and CRISPR-Cas9. *Stem Cell Reports* **4**, 143–154 (2015).
6. Ran, F. A. *et al.* In vivo genome editing using Staphylococcus aureus Cas9. *Nature* **520**, 186–191 (2015).
7. Kim, E. *et al.* In vivo genome editing with a small Cas9 orthologue derived from Campylobacter jejuni. *Nat Commun* **8**, (2017).
8. Nowak, C. M., Lawson, S., Zerez, M. & Bleris, L. Guide RNA engineering for versatile Cas9 functionality. *Nucleic Acids Res* **44**, 9555–9564 (2016).
9. Hou, Z. *et al.* Efficient genome engineering in human pluripotent stem cells using Cas9 from Neisseria meningitidis. *Proc Natl Acad Sci U S A* **110**, 15644–15649 (2013).
10. Kleinstiver, B. P. *et al.* Engineered CRISPR-Cas9 nucleases with altered PAM specificities. *Nature* **523**, 481–485 (2015).
11. Hirano, H. *et al.* Structure and Engineering of Francisella novicida Cas9. *Cell* **164**, 950–961 (2016).
12. Chen, F. *et al.* Targeted activation of diverse CRISPR-Cas systems for mammalian genome editing via proximal CRISPR targeting. *Nat Commun* **8**, (2017).
13. Müller, M. *et al.* Streptococcus thermophilus CRISPR-Cas9 systems enable specific editing of the human genome. *Molecular Therapy* **24**, 636–644 (2016).
14. Zetsche, B. *et al.* Cpf1 Is a Single RNA-Guided Endonuclease of a Class 2 CRISPR-Cas System. *Cell* **163**, 759–771 (2015).
15. Tu, M. *et al.* A new lease of life': FnCpf1 possesses DNA cleavage activity for genome editing in human cells. *Nucleic Acids Res* **45**, 11295–11304 (2017).
16. Teng, F. *et al.* Repurposing CRISPR-Cas12b for mammalian genome engineering. *Cell Discov* **4**, (2018).
17. Strecker, J. *et al.* Engineering of CRISPR-Cas12b for human genome editing. *Nat Commun* **10**, (2019).
18. Cox, D. B. T. *et al.* RNA editing with CRISPR-Cas13. *Science* **358**, 1019–1027 (2017).
19. Konermann, S. *et al.* Transcriptome Engineering with RNA-Targeting Type VI-D CRISPR Effectors. *Cell* **173**, 665-676.e14 (2018).
20. Liu, J. J. *et al.* CasX enzymes comprise a distinct family of RNA-guided genome editors. *Nature* **566**, 218–223 (2019).
21. Harrington, L. B. *et al.* Programmed DNA destruction by miniature CRISPR-Cas14 enzymes. *Science* **362**, 839–842 (2018).
22. Ono, H., Kawasaki, S. & Saito, H. Orthogonal Protein-Responsive mRNA Switches for Mammalian Synthetic Biology. *ACS Synth Biol* **9**, 169–174 (2020).
