# Supplementary material for: Programmable mammalian translational modulators by CRISPR-associated proteins
Source: Nat Commun. 2023 Apr 19;14:2243. doi: 10.1038/s41467-023-37540-7 (PMC10115826; doi:10.1038/s41467-023-37540-7)
Supplement: Supplementary file 2 — Reporting Summary [file 41467_2023_37540_MOESM2_ESM.pdf]

nature portfolio

Corresponding author(s): Hirohide Saito, Shunsuke Kawasaki

Last updated by author(s): 2023 March 10

Reporting Summary

Nature Portfolio wishes to improve the reproducibility of the work that we publish. This form provides structure for consistency and transparency in reporting. For further information on Nature Portfolio policies, see our Editorial Policies and the Editorial Policy Checklist.

Statistics

For all statistical analyses, confirm that the following items are present in the figure legend, table legend, main text, or Methods section.

n/a	Confirmed	
☐	☒	The exact sample size (*n*) for each experimental group/condition, given as a discrete number and unit of measurement
☐	☒	A statement on whether measurements were taken from distinct samples or whether the same sample was measured repeatedly
☐	☒	The statistical test(s) used AND whether they are one- or two-sided<br>*Only common tests should be described solely by name; describe more complex techniques in the Methods section.*
☒	☐	A description of all covariates tested
☐	☒	A description of any assumptions or corrections, such as tests of normality and adjustment for multiple comparisons
☐	☒	A full description of the statistical parameters including central tendency (e.g. means) or other basic estimates (e.g. regression coefficient) AND variation (e.g. standard deviation) or associated estimates of uncertainty (e.g. confidence intervals)
☐	☒	For null hypothesis testing, the test statistic (e.g. *F*, *t*, *r*) with confidence intervals, effect sizes, degrees of freedom and *P* value noted<br>*Give P values as exact values whenever suitable.*
☒	☐	For Bayesian analysis, information on the choice of priors and Markov chain Monte Carlo settings
☒	☐	For hierarchical and complex designs, identification of the appropriate level for tests and full reporting of outcomes
☐	☒	Estimates of effect sizes (e.g. Cohen's *d*, Pearson's *r*), indicating how they were calculated

Our web collection on statistics for biologists contains articles on many of the points above.

Software and code

Policy information about availability of computer code

Data collection	Cell images were obtained by Cytell Cell Imaging System Version 3.6.7.19 or CQ1 Software Version 1.07.01.01 (Yokogawa Electric Corporation). Flow cytometry data were obtained by CFlow Plus Software Version 1.0.227.4 or CSampler Software Version 1.0.264.21.
Data analysis	Statistical analysis and graphs were made by R, Python3.0, and Excel. Original phylogenetic tree was made by Multiple Sequence Comparison by Log- Expectation (MUSCLE: https://www.ebi.ac.uk/Tools/msa/muscle/). FlowJo version 10.5.3 was used for the flow cytometry analysis. TIFF files of the captured fluorescent cell images were analyzed using ImageJ version 1.53. The custom script for the fluorescent imaging analysis is available at (https://github.com/yfujita-skgcat/imageJ-cell-macros/blob/main/Calc_Each_Cell_Relative_Intensity.ijm). The custom script for MFE calculation and orthogonality validation is available at (https://github.com/Shunsuke-1994/CaRTRIDGE).

For manuscripts utilizing custom algorithms or software that are central to the research but not yet described in published literature, software must be made available to editors and reviewers. We strongly encourage code deposition in a community repository (e.g. GitHub). See the Nature Portfolio guidelines for submitting code & software for further information.

Data

Policy information about availability of data

All manuscripts must include a data availability statement. This statement should provide the following information, where applicable:

- Accession codes, unique identifiers, or web links for publicly available datasets
- A description of any restrictions on data availability
- For clinical datasets or third party data, please ensure that the statement adheres to our policy

All the data supporting this study are available within the main text and the Supplementary Information file. Source data are provided with this paper.

Human research participants

Policy information about studies involving human research participants and Sex and Gender in Research.

Reporting on sex and gender	N/A
Population characteristics	N/A
Recruitment	N/A
Ethics oversight	N/A

Note that full information on the approval of the study protocol must also be provided in the manuscript.

Field-specific reporting

Please select the one below that is the best fit for your research. If you are not sure, read the appropriate sections before making your selection.

☒ Life sciences ☐ Behavioural & social sciences ☐ Ecological, evolutionary & environmental sciences

For a reference copy of the document with all sections, see nature.com/documents/nr-reporting-summary-flat.pdf

Life sciences study design

All studies must disclose on these points even when the disclosure is negative.

Sample size	All data had at least 3 biological replicates (n>=3) in order to reduce the chances that results are due to variability or external factors.
Data exclusions	No data were excluded.
Replication	All experiments were reliably reproduced in independent settings for at least three times.
Randomization	No randomized experiment was performed in this study.
Blinding	Investigators were not blinded because our study is not a subjective trial and the results presented here are purely based on objective description.

Reporting for specific materials, systems and methods

We require information from authors about some types of materials, experimental systems and methods used in many studies. Here, indicate whether each material, system or method listed is relevant to your study. If you are not sure if a list item applies to your research, read the appropriate section before selecting a response.

Materials & experimental systems

n/a	Involved in the study	
☒	☐	Antibodies
☐	☒	Eukaryotic cell lines
☒	☐	Palaeontology and archaeology
☒	☐	Animals and other organisms
☒	☐	Clinical data
☒	☐	Dual use research of concern

Methods

n/a	Involved in the study	
☒	☐	ChIP-seq
☐	☒	Flow cytometry
☒	☐	MRI-based neuroimaging

Eukaryotic cell lines

Policy information about cell lines and Sex and Gender in Research

Cell line source(s)	HEK293FT (invitrogen)<br>HeLa CCL2 (ATCC)<br>A549 and iPS cell line (1383D6) (RIKEN)
Authentication	The cell line has been used in the lab for over 3 years. Authentications were not performed during this study.
Mycoplasma contamination	Cell line has been routinely subjected to Lonza's MycoAlert assay during this research and tested negative for Mycoplasma contamination.
Commonly misidentified lines (See ICLAC register)	No commonly misidentified cell lines were used in the study.

Flow Cytometry

Plots

Confirm that:

☒ The axis labels state the marker and fluorochrome used (e.g. CD4-FITC).

☒ The axis scales are clearly visible. Include numbers along axes only for bottom left plot of group (a 'group' is an analysis of identical markers).

☒ All plots are contour plots with outliers or pseudocolor plots.

☒ A numerical value for number of cells or percentage (with statistics) is provided.

Methodology

Sample preparation	Cultured and transfected cells were washed once with pre-warmed PBS, trypsinized with 0.25% Trypsin-EDTA at 37°C for 5 minutes, and resuspended in pre-warmed cell culture medium to trypsinization. The cells were filtered through a mesh filter before flow cytometric analysis.
Instrument	BD Accuri C6 flow cytometer (BD Biosciences)
Software	CFlow Plus software Version 1.0.227.4 or CSampler Software Version 1.0.264.21 were used to control the flow cytometer and collect the flow cytometry data. FlowJo version 10.5.3 was used to analyze the flow cytometry data.
Cell population abundance	At least 10,000 living cells were analyzed.
Gating strategy	Live cells were gated in the forward scatter (FSC) versus side scatter (SSC) plot to eliminate debris.<br>The remaining P1-positive events were plotted in the FL1-A (EGFP expression, Y-axis) versus FL4-A (iRFP670 expression, X-axis) and events on each axis line were ruled out by the gate (FL4-A, FL1-A subset). Then, the FL4-A subset gate was generated based on untransfected samples and thereby transfection-positive (FL4-positive) populations were defined.

☒ Tick this box to confirm that a figure exemplifying the gating strategy is provided in the Supplementary Information.
